# Integrative Bulk and Single-Cell Transcriptomic Profiling Reveals Oxidative Stress-Related Genes and Potential Therapeutic Targets in Osteoarthritis

**DOI:** 10.1155/mi/1240226

**Published:** 2025-10-10

**Authors:** Jinhui Peng, Jinzhong Chen, Duan Gao, Bowei Liang, Zongquan Huang, Bo Xiong, Shuheng Zhou, Guanghai Tan, Zhihui Zhong, Xianghong Zeng

**Affiliations:** ^1^Department of Minimally Invasive Spine, Yulin Orthopedic Hospital of Integrated Traditional Chinese and Western Medicine, Yulin, Guangxi, China; ^2^Department of Joint Surgery and Geriatric Orthopedics, Affiliated Hospital of Youjiang Medical University for Nationalities, Baise, Guangxi, China; ^3^Department of Anesthesiology, Zhongshan Hospital of Traditional Chinese Medicine Affiliated to Guangzhou University of Traditional Chinese Medicine, Zhongshan, Guangdong, China; ^4^Hospital Administration Office, The First Hospital of Yulin, Yulin, Guangxi, China; ^5^Hospital Administration Office, Yulin Orthopedic Hospital of Integrated Traditional Chinese and Western Medicine, Yulin, Guangxi, China; ^6^Guangdong Pharmaceutical University, Guangzhou, Guangdong, China; ^7^Department of Internal Medicine, Yulin Orthopedic Hospital of Integrated Traditional Chinese and Western Medicine, Yulin, Guangxi, China; ^8^Clinical Medical College of Tianjin Medical University, Tianjin, China; ^9^Stem Cell and Regenerative Medicine Center, Gaozhou People's Hospital, Maoming, Guangdong, China; ^10^Department II of Hand Surgery, Yulin Orthopedic Hospital of Integrated Traditional Chinese and Western Medicine, Yulin, Guangxi, China

**Keywords:** bioinformatics, FOS, molecular docking, osteoarthritis, oxidative stress, single-cell RNA sequencing

## Abstract

Osteoarthritis (OA) is increasingly recognized as a degenerative joint disease that leads to a serious problem of public health, yet the underlying molecular mechanisms remain incompletely understood. In this study, we integrated bulk and single-cell RNA sequencing (scRNA-seq) datasets from the Gene Expression Omnibus (GEO) to systematically investigate oxidative stress–related genes and pathways in OA. Gene set enrichment analysis (GSEA) revealed significant activation of oxidative stress signaling in OA cartilage tissues, with 58 differentially expressed oxidative stress-related genes identified. Subsequent LASSO regression analysis highlighted seven diagnostic genes (STC2, LSP1, COL6A1, FOS, SELENON, TP53, and HSPA8), which demonstrated robust diagnostic performance in both training and validation cohorts. Single-cell analysis further revealed cell-type-specific differences in oxidative stress activity, with homeostatic chondrocytes (HomCs) exhibiting the highest pathway scores. Among the identified genes, FOS emerged as a hub regulator, showing elevated expression in HomCs from OA samples and strong associations with immune infiltration and proinflammatory pathways. Functional assays demonstrated that FOS knockdown significantly attenuated IL-1β-induced oxidative stress, apoptosis, and inflammatory cytokine (interleukin-6 [IL-6] and tumor necrosis factor-alpha [TNF-α]) release in chondrocytes. Furthermore, molecular docking and dynamics simulations identified ursolic acid (UA) as a stable small-molecule FOS binder, and in vitro experiments confirmed its inhibitory effects on oxidative stress and inflammation, comparable to FOS silencing or pharmacological inhibition. Collectively, our findings suggest that oxidative stress–related genes, particularly FOS, play a central role in OA pathogenesis by linking redox imbalance to immune dysregulation and chondrocyte injury, and highlight UA as a potential therapeutic candidate for OA management.

## 1. Introduction

Osteoarthritis (OA) is the most common chronic joint disease, affecting more than 300 million people worldwide and representing a leading cause of chronic pain and disability, particularly among the elderly [1]. It is a leading cause of pain and disability, typically affecting weight-bearing joints such as the knees, hips, and spine [2]. In the United States alone, OA-related healthcare costs and productivity losses exceed $486 billion annually [3], while globally, it ranks among the top 10 causes of years lived with disability in high-income countries [4]. The pathogenesis of OA is complex and involves a combination of mechanical, biochemical, and inflammatory factors. Traditionally viewed as a degenerative cartilage disease, OA is now recognized as a multifactorial disorder involving the entire joint structure. Key pathological features include progressive articular cartilage degradation, synovial inflammation, subchondral bone remodeling, osteophyte formation, and alterations in the meniscus and ligaments [5]. Molecular mechanisms contributing to OA pathogenesis include increased expression of proinflammatory cytokines (e.g., IL-1β, tumor necrosis factor-alpha [TNF-α]), oxidative stress, extracellular matrix (ECM) breakdown by matrix metalloproteinases (MMPs), and dysregulated chondrocyte metabolism [6, 7]. These processes contribute to joint structure deterioration and functional decline, underscoring the importance of understanding OA's complex biology for developing targeted therapies.

Oxidative stress, resulting from an imbalance between reactive oxygen species (ROS) production and antioxidant defenses, has emerged as a key contributor to cartilage degradation, synovial inflammation, and aberrant chondrocyte signaling in OA, as evidenced by elevated oxidative stress biomarkers in patient synovial fluid and serum [8–10]. Oxidative stress refers to a pathological condition arising from an imbalance between the production of ROS and the antioxidant defense systems of the body, leading to cellular and extracellular damage [11]. Elevated levels of ROS have been detected in the synovial fluid and cartilage of OA patients, where they contribute to chondrocyte apoptosis, ECM degradation, and inflammatory responses [12, 13]. ROS can activate catabolic signaling pathways, such as NF-κB and MAPK, thereby upregulating the expression of MMPs and proinflammatory cytokines like IL-1β and TNF-α, which further promote cartilage destruction [14]. Moreover, oxidative stress impairs mitochondrial (mt) function and antioxidant enzyme activity in chondrocytes, exacerbating joint tissue damage [15]. The accumulation of oxidative damage is also associated with age-related OA, as the aging joint environment becomes more susceptible to redox imbalance and chronic inflammation [16]. Targeting oxidative stress has thus emerged as a promising therapeutic strategy in OA management. Targeting oxidative stress offers a promising avenue for OA treatment. Various antioxidants have been tested in experimental models and clinical trials with varying degrees of success. Natural compounds such as resveratrol, curcumin, and quercetin exhibit both antioxidant and anti-inflammatory properties, making them attractive candidates for OA therapy [17]. Pharmacological agents such as N-acetylcysteine (NAC) and mitoquinone (MitoQ) specifically target intracellular and mt ROS, respectively, and have shown protective effects on cartilage in vitro and in vivo [18]. Furthermore, gene therapy approaches that enhance antioxidant enzyme expression or Nrf2 activation are currently under investigation and may provide long-term protection against oxidative joint damage. However, challenges remain in delivering these therapies effectively and selectively to affected joints.

In this study, we comprehensively integrated bulk RNA-sequencing and single-cell transcriptomic data to investigate the activation patterns of genes involved in the oxidative stress signaling pathway in OA. By leveraging differential gene expression analysis and functional enrichment approaches, we identified key diagnostic markers associated with oxidative stress. We further explored the potential biological pathways and immune cell infiltration patterns regulated by these genes to better understand their roles in the pathogenesis of OA. Among the identified candidates, FOS emerged as a critical hub gene, warranting further in-depth characterization at the single-cell level. We examined its expression across cellular subsets, evaluated its functional implications, and investigated its downstream signaling activity. Moreover, to assess its potential as a therapeutic target, we conducted virtual screening and molecular docking analyses to identify small-molecule compounds with high binding affinity to FOS, thereby offering new insights into possible treatment strategies for OA.

## 2. Methods and Materials

### 2.1. Data Acquisition and Organization

We obtained three transcriptomic datasets associated with OA from the Gene Expression Omnibus (GEO, https://www.ncbi.nlm.nih.gov/gds) database for analysis. The GSE236924 dataset, used as a test set, includes seven healthy control samples and 89 OA tissue samples, while GSE82107, serving as a validation set, comprises seven healthy controls and 10 OA samples. Additionally, the single-cell RNA sequencing (scRNA-seq) dataset GSE169454 contains data from three healthy controls and four OA samples. All the tissues are cartilage tissues derived from the knee joint. To investigate oxidative stress-related mechanisms in OA, we retrieved relevant signaling pathways via the key word of “oxidative stress” from the Molecular Signatures Database (MsigDB, https://www.gsea-msigdb.org/gsea/msigdb/index.jsp), including GOBP_CELLULAR_RESPONSE_TO_OXIDATIVE_STRESS, GOBP_NEGATIVE_REGULATION_OF_CELLULAR_RESPONSE_TO_OXIDATIVE_STRESS, GOBP_NEGATIVE_REGULATION_OF_RESPONSE_TO_OXIDATIVE_STRESS, GOBP_REGULATION_OF_CELLULAR_RESPONSE_TO_OXIDATIVE_STRESS, GOBP_RESPONSE_TO_OXIDATIVE_STRESS in the C5 ontology gene sets, for ROS are the main perpetrators of oxidative stress, HALLMARK_REACTIVE_OXYGEN_SPECIES_PATHWAY in the hallmark gene sets were also collected.. After consolidating these gene sets, we identified a total of 419 unique oxidative stress-related genes for subsequent analyses.

### 2.2. Analysis of Differentially Expressed Genes (DEGs) Associated With Oxidative Stress

Expression profiles of oxidative stress-related genes were extracted and analyzed using the “limma” package to identify DEGs between OA and healthy control samples. Genes with an adjusted *p*-value < 0.05 and an absolute log_2_ fold change (|log_2_FC|) > 1 were considered significantly differentially expressed.

### 2.3. Pathways Annotation and Protein–Protein Interaction (PPI) Analysis

Gene Ontology (GO) and Kyoto Encyclopedia of Genes and Genomes (KEGG) enrichment analyses were conducted on the DEGs to explore their functional implications. Additionally, gene set enrichment analysis (GSEA) was performed for each key gene to assess associated biological pathways, while the irGSEA algorithm was employed to calculate enrichment scores of hallmark gene sets in the MsigDB at the single-cell level [19]. To explore the functional associations of DEGs between FOS-high and FOS-low homeostatic chondrocytes (HomCs), we constructed a PPI network using the STRING database (https://string-db.org/). The list of potential target genes was uploaded into STRING, with *Homo sapiens* selected as the reference species. The minimum required interaction score was set to 0.4 (medium confidence) to balance sensitivity and specificity. Both experimentally validated interactions and predicted associations (including text mining, coexpression, database annotation, and protein homology) were included. The resulting interaction network was visualized and further analyzed using Cytoscape (version 3.8.2), where key nodes were identified based on degree centrality (cytoHubba plugin).

### 2.4. Identification of Diagnostic Genes via LASSO Cox Regression Analysis

To identify diagnostic genes associated with OA among the differentially expressed oxidative stress-related genes, we performed LASSO Cox regression analysis. To prevent model overfitting and enhance robustness, 10-fold cross-validation was applied to determine the optimal penalty parameter and calculate gene coefficients. The diagnostic performance of the selected genes was evaluated using receiver operating characteristic (ROC) curve analysis. Furthermore, to facilitate clinical prediction of OA, a nomogram model was constructed based on the oxidative stress-related genes identified by the LASSO method using regression modeling techniques, providing an intuitive tool for individualized risk assessment.

### 2.5. Analysis of Immune-Infiltrating Cells

We applied the CIBERSORT algorithm to estimate the relative proportions of 22 distinct immune cell types within each sample, enabling the assessment of immune cell infiltration in OA tissues. In parallel, oxidative stress signaling pathway scores were calculated for each sample to evaluate their association with immune cell abundance. Furthermore, Spearman correlation analyses were performed to explore the relationship between the expression levels of candidate oxidative stress-related genes and the degree of immune cell infiltration.

### 2.6. Analysis of scRNA-Seq Data

ScRNA-seq data were processed using the Seurat v4.0 package. To integrate data from multiple samples, we applied the Harmony algorithm, effectively correcting for batch effects and enhancing downstream analysis consistency. Quality control measures were implemented to exclude low-quality cells, specifically removing those with a mt or ribosomal (rb) gene content exceeding 20%, cells expressing fewer than 200 or more than 10,000 genes, and those with a total gene expression count below 60,000. The remaining high-quality cells were normalized using the “NormalizeData” function. Subsequently, we identified the top 2000 highly variable genes with the “FindVariableFeatures” function and performed principal component analysis (PCA), retaining the top 20 principal components (PCs) for downstream dimensionality reduction and clustering. After assessing the PCs with the JackStraw and Elbow methods, cell clustering was carried out using the “FindNeighbors” and “FindClusters” functions. Upon determining the optimal clustering resolution, the UMAP algorithm was employed to project the data into a low-dimensional space, facilitating the visualization of distinct cell populations. Then the identified clusters were manually annotated by referencing well-established cell type-specific marker genes from previously published literature [20, 21]. To explore the role of oxidative stress-related genes in OA patients and chondrocytes, we utilized the “AddModuleScore” function to calculate the module scores for oxidative stress-associated gene sets in single-cell data.

### 2.7. Intercellular Communication Analysis

We utilized the CellChat package to construct a CellChat object and configured the ligand–receptor interaction database (CellChatDB.human). The scRNA seq expression profiles were preprocessed to serve as input for inferring both incoming and outgoing intercellular signaling. Subsequently, communication probabilities at the pathway level were inferred by calculating the interaction likelihood of all ligand-receptor pairs associated with each signaling pathway. In addition, interaction networks were generated to visualize and quantify the number and strength of intercellular communications among different cell types.

### 2.8. Searching for Potential Drugs for Molecular Docking and Molecular Dynamics Simulation

We searched for potential targeted drugs for the identified genes using the BATMAN database (http://bionet.ncpsb.org.cn/batman-tcm/#/home), with the confidence score set to the default parameters [22]. The resulting network was then imported into the Cytoscape software for visualization. We obtained the crystal structure of the target protein from the AlphaFold Protein Structure Database (https://alphafold.ebi.ac.uk/), which provides high-confidence predicted protein models based on deep learning [23]. Meanwhile, the 3D structure files (SDF format) of the selected small-molecule compounds were downloaded from the PubChem database (https://pubchem.ncbi.nlm.nih.gov/). These molecular structures were subsequently input into the HDOCK platform (http://hdock.phys.hust.edu.cn/) to perform molecular docking analysis, aiming to predict the binding affinity and interaction modes between the compounds and the target protein [24]. Finally, the PyMOL 3.1 software was used to visualize the ligand-protein docking [24]. Molecular dynamics simulations were conducted using GROMACS 2022. The amber14sb force field was applied for the receptor and GAFF2 for the ligand, with parameters generated by pdb2gmx and AutoFF [25]. The system was solvated in a cubic TIP3P water box (1 nm margin) and neutralized with counter ions. Electrostatic interactions were treated with the Particle Mesh Ewald (PME) method (1 nm cutoff), and covalent bonds were constrained using the SHAKE algorithm with a 1 fs integration step. The system was energy-minimized and equilibrated before production runs. Simulations were performed under NPT conditions at 310 K for 100 ns. Structural analyses included root mean square deviation (RMSD), root mean square fluctuation (RMSF), hydrogen bonding, radius of gyration (Rg), and solvent-accessible surface area (SASA), calculated using standard GROMACS utilities.

### 2.9. RNA Isolation and Quantitative Polymerase Chain Reaction (Q-PCR)

Total RNA was extracted from the harvested cells using a commercially available RNA isolation kit (#EZB-RN001-plus, EZBioscience), following the manufacturer's protocol. The quality and concentration of RNA were assessed using a NanoDrop 2000 spectrophotometer (Thermofisher). Reverse transcription was performed using a cDNA synthesis kit (#R323-01, Vazyme), and quantitative PCR was carried out using SYBR Green Master Mix on a real-time PCR system (ABI 7500 Real-Time PCR System, ThermoFisher) to quantify gene expression levels. The housekeeping gene GAPDH was used as an internal control. The sequences of the primers are listed in Supporting Information 1: Table S1.

### 2.10. Cell Culture and Treatment

The SW1353 cell line (CELLCOOK, Guangzhou, China) was cultured in L-15 medium supplemented with 10% fetal bovine serum (FBS, Gibco) at 37 °C in an atmosphere containing 5% CO_2_ and 95% air. When the cells (5 × 10^5^) in the six-well plate reached ~80% confluence, they were subjected to IL-1β (20 ng/mL, Chamot Biotechnology, #CM002-5HP) to build a cell model. After 24 h of treatment, the cells were harvested for further analysis. Furthermore, the potential FOS targeted drug ursolic acid (UA; 5 μM, Targetmol, #T0722) was introduced for evaluating its function in release. SR11302 (5 μM, MedChemExpress, #HY-15870) was used as a positive control to compare the effects of small molecules.

### 2.11. Small Interfering RNA Transfection

To investigate the potential effect of FOS on OA, we knocked down FOS in the SW1353 cells. The commercialized siRNA was synthesized by Genepharma Company. The sequence of FOS: CUCCAGUGCCAACUUCAUUTT (sense 5′–3′), AAUGAAGUUGGCACUGGAGTT (antisense 5′-3′), and the sequence of the negative control: UUCUCCGAACGUGUCACGUTT (sense 5′–3′), ACGUGACACGUUCGGAGAATT (antisense 5′-3′). The specific operation should be carried out according to the instructions. In brief, 5 × 10^5^ cells per well, when the cell fusion degree is 70%–80%, add the mixture of accompanying transfection lipid and siRNA, cultivate for the specified time, 4 h, remove the culture medium, and continue to cultivate for 24 h.

### 2.12. Cell Apoptosis

Cell apoptosis was assessed using the annexin V-FITC/7-aminoactinomycin D (7-AAD) double-staining method (Elabscience, #E-CK-A212) followed by flow cytometry. Briefly, cells were harvested, washed twice with cold phosphate-buffered saline (PBS), and resuspended in binding buffer at a concentration of 5 × 10^5^ cells/mL. Subsequently, 5 µL of annexin V-FITC and 5 µL of 7-AAD were added to 100 µL of the cell suspension, followed by incubation in the dark at room temperature for 15 min. After staining, 400 µL of binding buffer was added to each sample, and apoptotic cells were immediately analyzed using a flow cytometer (BD Bioscience). The proportion of early apoptotic (annexin V^+^/7-AAD^−^) and late apoptotic or necrotic (annexin V^+^/7-AAD^+^) cells was quantified. Data were analyzed using FlowJo software, and representative results were derived from three independent experiments.

### 2.13. Intracellular ROS Measurement

Intracellular ROS levels were measured using the fluorescent probe 2′, 7′-dichlorodihydrofluorescein diacetate (DCFH-DA, Beyotime, #S0033S-1). Cells were seeded in 12-well plates and treated as indicated. After treatment, cells were washed twice with PBS and incubated with 10 μM DCFH-DA diluted in serum-free medium at 37 °C for 30 min in the dark. Following incubation, cells were washed three times with PBS to remove excess probe. The fluorescence intensity, which correlates with intracellular ROS levels, was immediately quantified by flow cytometry with excitation at 488 nm and emission at 525 nm. All measurements were performed in triplicate, and mean fluorescent intensity data were analyzed using FlowJo software.

### 2.14. Enzyme-Linked Immunosorbent Assay (ELISA)

The concentrations of interleukin-6 (IL-6, jymbio, #JYM0140Hu) and TNF-α (jymbio, #JYM0110Hu) in cell culture supernatants were determined using commercially available ELISA kits according to the manufacturer's instructions. Briefly, samples and standards were added to 96-well microplates precoated with specific antibodies against IL-6 or TNF-α and incubated at 37 °C for 1–2 h. After washing to remove unbound substances, horseradish peroxidase (HRP)-conjugated detection antibodies were added and incubated, followed by another series of washes. Substrate solution (TMB) was then added for color development, and the reaction was terminated with the stop solution. The optical density (OD) was measured at 450 nm using a microplate reader.

### 2.15. Statistical Analysis

All statistical analyses were conducted using R software (version 4.0.3) and GraphPad Prism (version 8.1). Comparisons of pathway enrichment scores and immune cell infiltration levels between groups were performed using the Wilcoxon rank-sum test. Correlation analyses were carried out using Spearman's rank correlation coefficient. Gene expression levels were quantified using the 2^–ΔΔCt^ method. The comparison between the two groups was conducted using the *t*-test method. For comparisons among multiple groups, the one-way analysis of variance (ANOVA) was applied. Data are presented as mean ± standard deviation (SD), and a *p*-value < 0.05 was considered statistically significant.

## 3. Results

### 3.1. The Oxidative Stress Signaling Pathway Was Enriched in OA

**Figure 1:**
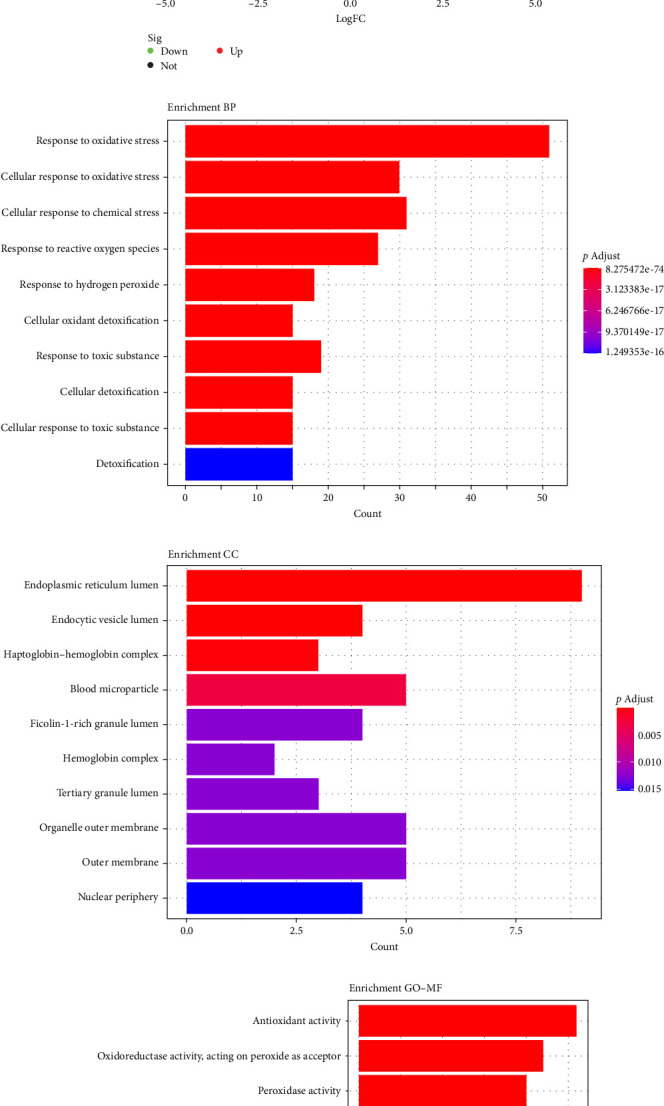
Identification of oxidative stress-related DEGs. (A) GSEA analysis of the enrichment of the oxidative stress signaling pathway in OA. (B) Comparison of oxidative stress scores between OA and control samples. (C) Volcano plot displaying differentially expressed genes. (D–F) Biological process (BP), cell component (CC), and molecular function (MF) of DEGs were analyzed via GO, respectively. (G) DEGs were analyzed via KEGG. *⁣*^*∗∗*^*p*  < 0.01.

To investigate the involvement of oxidative stress pathways, we first performed GSEA on the bulk RNA sequencing data from the GSE236924 dataset. The analysis revealed a marked enrichment of oxidative stress-related gene signatures in OA samples (*p*=0.000169, Figure 1A). Comparison of the scores between the two groups revealed that the scores of OA tissues were much higher than those of control tissues (*p*=0.0079, Figure 1B). Subsequently, the differential expression of 419 oxidative stress-related genes was analyzed between the two groups. As illustrated in the volcano plot, a total of 58 DEGs were identified, including 25 downregulated and 33 upregulated genes in OA tissues (Figure 1C). In addition, GO and KEGG pathway enrichment analyses were conducted on the DEGs. In the GO biological process (BP) category, the genes were primarily enriched in response to oxidative stress and cellular response to oxidative stress (Figure 1D). For the cellular component (CC) category, enrichment was observed in the membrane- and lumen-associated structures, including the endoplasmic reticulum (ER) lumen, endocytic vesicle lumen, and outer membrane (Figure 1E). Within the molecular function (MF) category, the genes were mainly associated with antioxidant activity, oxidoreductase activity, and peroxidase activity (Figure 1F). KEGG pathway analysis revealed significant enrichment in the longevity-regulating pathway, and inflammatory-related pathways such as the TNF signaling pathway, PI3K-Akt signaling pathway, and IL-17 signaling pathway (Figure 1G). The above results suggest that the oxidative stress signaling pathway may be activated in OA.

### 3.2. Distribution of Oxidative Stress Signaling Pathways in Different Cell Types

**Figure 2:**
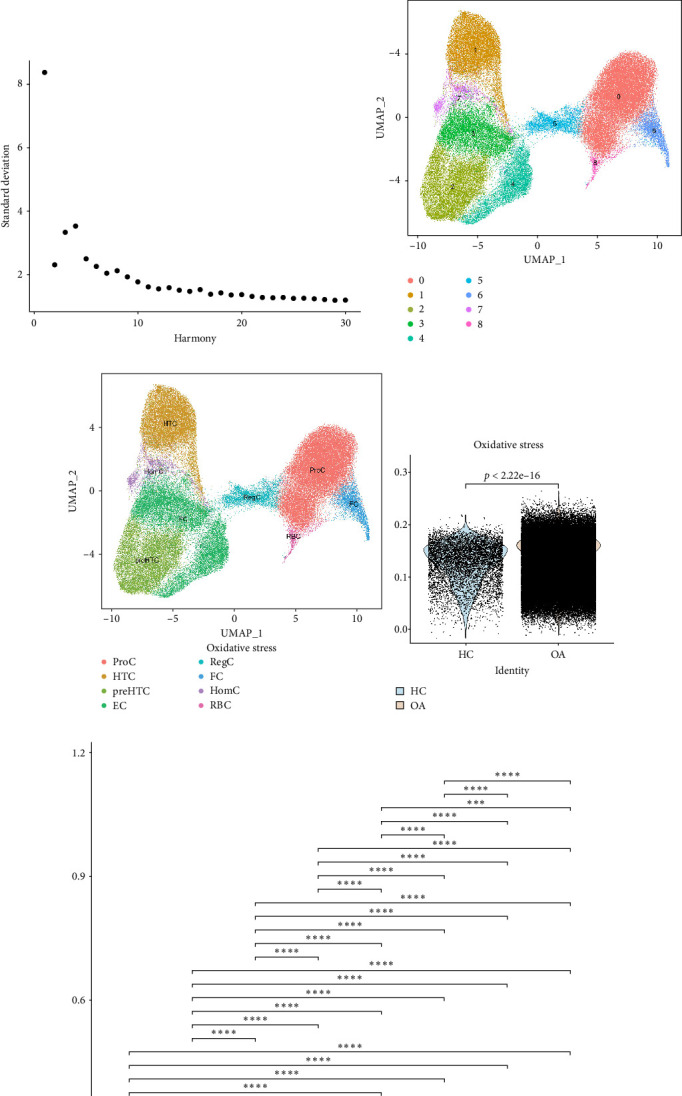
Analysis of oxidative stress signal distribution among distinct cell populations at the single-cell level. (A) Filtering of single-cell RNA-seq data. (B) Batch effect correction was performed using the Harmony algorithm. (C) The top 2000 highly variable genes. (D) Identification of the optimal standard deviation. (E, F) Cell clustering and annotation of dataset GSE169454. (G) Comparison of oxidative stress scores between OA and control cells. (H) Comparison of oxidative stress scores between different cell populations. (I) Comparative analysis of oxidative stress scores between different cell types in OA and control groups. *⁣*^*∗*^*p*  < 0.05, *⁣*^*∗∗*^*p*  < 0.01, *⁣*^*∗∗∗*^*p*  < 0.001, *⁣*^*∗∗∗∗*^*p*  < 0.0001.

By applying quality control thresholds based on nFeature, nCount, and the percentages of mt and rb gene expression of GSE169454, a total of 68,073 high-quality cells were retained for downstream analysis (Figure 2A). To eliminate batch effects across samples, the Harmony package was employed for integration and correction (Figure 2B). The top 2000 highly variable genes were identified (Figure 2C). Based on the elbow plot, the optimal SD value for downstream analysis was determined to be 8 (Figure 2D). At a resolution parameter of 0.3, a total of 9 distinct cell clusters were identified (Figure 2E). A total of 9 cell clusters were identified. The distribution of cells across clusters was as follows: Cluster 0 contained the largest number of cells (38,327), followed by Cluster 1 (18,895), Cluster 2 (15,254), Cluster 3 (12,841), Cluster 4 (8516), Cluster 5 (4848), Cluster 6 (4028), Cluster 7 (2629), and Cluster 8 (1062). Based on reference genes reported in the literature, we annotated eight distinct cell populations: proliferative chondrocytes (ProCs), hypertrophic chondrocytes (HTCs), pre-HTCs (preHTCs), effector chondrocytes (ECs), fibrocartilage chondrocytes (FCs), regulatory chondrocytes (RegCs), HomCs, and red blood cells (RBCs) (Figure 2F). To assess the activity of oxidative stress signaling pathways across different cell types, we utilized the “AddModuleScore” function in the Seurat package to calculate module scores based on the expression levels of oxidative stress-related gene sets. The results showed that the oxidative stress signaling pathway score in tumor tissue cells was significantly increased compared with normal tissue cells (*p*  < 0.0001, Figure 2G). Significant differences in oxidative stress scores were observed across cell types, with HomCs exhibiting the highest scores, while ProCs and FCs displayed comparatively lower scores (Figure 2H). Further comparative analysis revealed that oxidative stress signaling pathway scores were elevated in OA samples for HTC, EC, RegC, HomC, and RBC populations, whereas ProC, preHTC, and FC populations exhibited lower scores (Figure 2I). These findings indicate that the activation patterns of oxidative stress signaling pathways vary across different cell types.

### 3.3. Identification of Seven Diagnostic Genes Related to Oxidative Stress in OA

**Figure 3:**
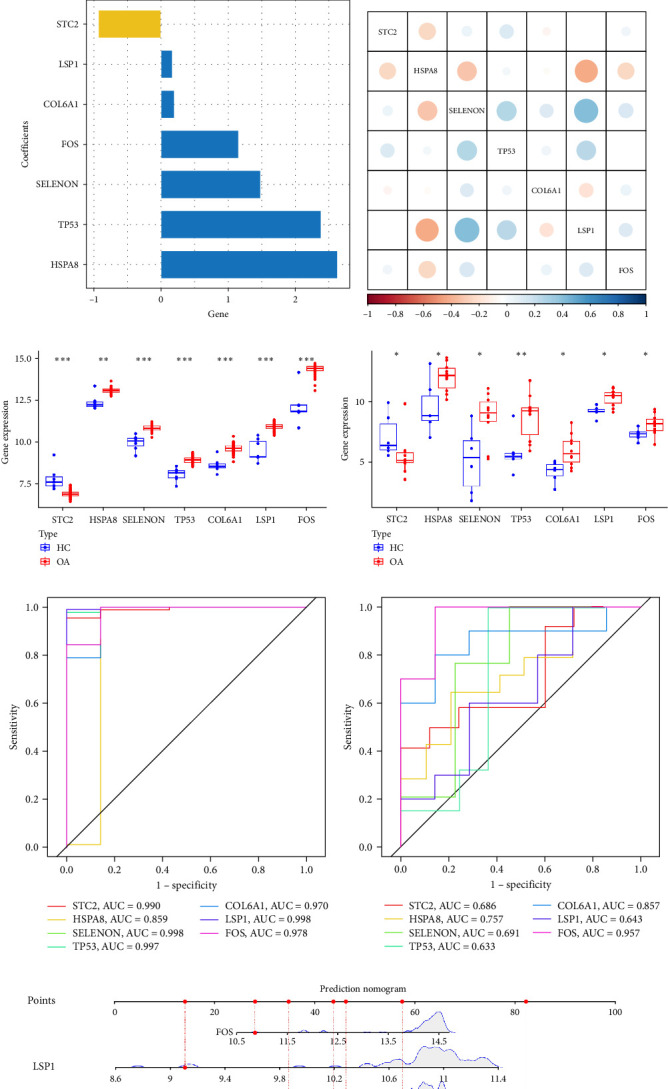
Identification of key oxidative stress genes in OA. (A) LASSO regression analysis of oxidative stress-related DEGs. (B) Bar Plot representing the coefficient values of key genes. (C) Correlation analysis among the seven key genes. Red represents positive correlation, with deeper shades indicating stronger positive correlation. Blue represents negative correlation, with deeper shades indicating stronger negative correlation. (D, E) Comparison of key gene expression between OA and control groups in the training and validation sets. (F, G) ROC curves of key genes in the training and validation sets. (H) Development of a clinical Nomogram tool. *⁣*^*∗*^*p*  < 0.05, *⁣*^*∗∗*^*p*  < 0.01, *⁣*^*∗∗∗*^*p*  < 0.001, *⁣*^*∗∗∗∗*^*p*  < 0.0001. HC, healthy control; OA, osteoarthritis.

To further identify potential biomarkers among oxidative stress-related DEGs in OA, we applied the LASSO algorithm with 10-fold cross-validation to select the most predictive key genes (Figure 3A). Seven genes, STC2, LSP1, COL6A1, FOS, SELENON, TP53, and HSPA8, were identified, among which all except STC2 exhibited positive coefficients, suggesting that these genes may serve as potential risk factors (Figure 3B). Correlation analysis among the seven genes revealed that LSP1 was strongly positively correlated with HSPA8 while exhibiting negative correlations with SELENON and TP53 (Figure 3C). Interestingly, among the seven identified genes, only STC2 was downregulated in OA tissues, while the remaining genes were upregulated; notably, this expression pattern was consistently observed in both the training and validation sets (Figure 3D,E). Subsequently, to evaluate the diagnostic performance of the seven identified genes in OA, ROC curve analysis was performed, and the corresponding AUC values were calculated to assess their sensitivity and specificity. In the training cohort, among the seven genes, HSPA8 exhibited the lowest AUC value, yet still reached 0.859 (95% CI: 0.792–0.868), indicating strong diagnostic performance (Figure 3F). In the validation set, although the predictive power of six genes, excluding FOS, declined slightly, all maintained AUC values above 0.63 (95% CI: 0.58–0.72), suggesting that their predictive performance remained stable and reliable (Figure 3G). A nomogram incorporating the seven specific genes was developed to predict the probability of OA, where each gene contributes a weighted score, and the cumulative total score is used to estimate an individual's overall risk of developing OA (Figure 3H).

### 3.4. Potential Pathways Involved in the Key Genes

**Figure 4:**
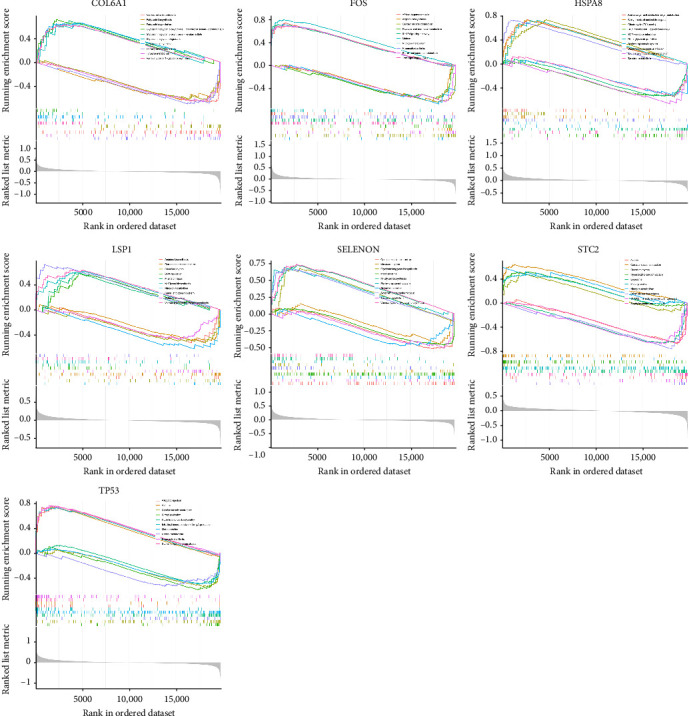
Potential pathways involving key genes identified by GSEA analysis.

To further investigate the potential roles of the key genes in distinguishing OA from control samples, we performed single-gene GSEA. This approach provided insight into the biological pathways associated with each gene's expression, revealing their mechanistic relevance in OA pathogenesis (Figure 4). In terms of downregulated pathways, COL6A1, FOS, LSP1, SELENON, and TP53 were all enriched in cardiac muscle contraction-related pathways, suggesting potential roles beyond the cardiovascular system, possibly in maintaining structural integrity in cartilage. COL6A1 was also associated with fatty acid metabolism, highlighting its possible involvement in lipid regulation and energy supply within the joint microenvironment. FOS was linked to mannose and sucrose metabolism, while HSPA8 was enriched in hypotaurine and tyrosine metabolism, pointing to roles in glycosylation, oxidative stress balance, and neurotransmitter-related processes. Additionally, LSP1, TP53, and SELENON showed enrichment in nitrogen metabolism, indicating relevance to amino acid turnover and stress responses. STC2 was uniquely associated with SNARE interactions in vesicular transport, potentially influencing matrix secretion and chondrocyte communication. As for the upregulated pathways, COL6A1, LSP1, and SELENON were significantly enriched in N-glycan biosynthesis, with COL6A1 showing a strong connection to glycosaminoglycan metabolism, critical for maintaining cartilage structure. FOS was implicated in IL-17 and TNF inflammatory pathways, key drivers of OA-related inflammation and matrix degradation. HSPA8 was associated with the tricarboxylic acid (TCA) cycle and amino sugar metabolism, reflecting its role in energy metabolism and ECM turnover. LSP1 and SELENON were also connected to the renin–angiotensin system, suggesting roles in vascular and immune modulation. Notably, STC2 showed enrichment in circadian rhythm and nitrogen metabolism pathways, often downregulated in other genes, indicating a distinct regulatory role. TP53 was linked to autoimmune diseases, reinforcing the connection between OA and immune dysregulation. We further analyzed the pathway activity scores based on the HALLMARK gene sets and observed that pathways, including KRAS_SIGNALING_UP, ALLOGRAFT_REJECTION, UV_RESPONSE_DN, INFLAMMATORY_RESPONSE, EPITHELIAL_MESENCHYMAL_TRANSITION, HEDGEHOG_SIGNALING, APICAL_SURFACE, PROTEIN_SECRETION, and NOTCH_SIGNALING were significantly upregulated in OA samples. In contrast, pathways such as FATTY_ACID_METABOLISM, MYOGENESIS, and HYPOXIA exhibited decreased activity compared to the normal group (Supporting Information 2: Figure S1A). Correlation analysis between key genes and HALLMARK pathway scores revealed distinct association patterns across the seven genes, indicating their diverse functional roles in OA (Supporting Information 2: Figure S1B). For instance, STC2 exhibited a negative correlation with the NOTCH_SIGNALING pathway, in contrast to the other genes, such as FOS, TP53, and COL6A1, which showed positive correlations with this pathway. This suggests that STC2 may exert an inhibitory or regulatory effect on pathways that are otherwise activated by other key genes in OA. Additionally, FOS and TP53 demonstrated strong positive correlations with hallmark pathways involved in APOPTOSIS and INFLAMMATORY_RESPONSE, highlighting their potential contributions to chondrocyte death and chronic synovial inflammation in OA. These correlations underscore the multifaceted regulatory landscape orchestrated by these genes and provide evidence that their combined activity may influence disease severity through distinct, yet partially overlapping, molecular pathways. The diversity in correlation patterns also suggests the possibility of subtype-specific gene-pathway interactions that may underlie the heterogeneity of OA pathology. These findings collectively suggest that the seven key genes contribute to OA through diverse pathways involving metabolism, inflammation, immunity, and matrix homeostasis.

### 3.5. Oxidative Stress Signal and the Identified Genes Involved in Immune Cell Infiltration

**Figure 5:**
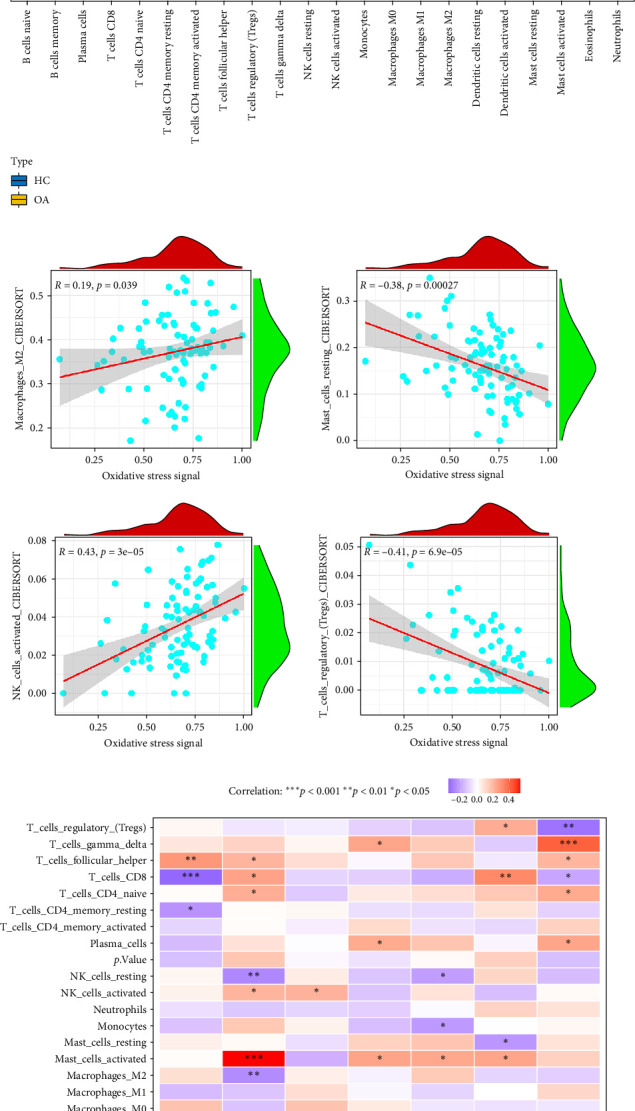
Oxidative stress correlates with immune cell infiltration in OA. (A) Differences in infiltration of immune cells between disease and control groups. (B) Infiltrating immune cell types are significantly associated with oxidative stress scores. (C) Analysis of the correlation between key gene infiltration in immune cells. Red represents positive correlation, with deeper shades indicating stronger positive correlation. Blue represents negative correlation, with deeper shades indicating stronger negative correlation. *⁣*^*∗*^*p*  < 0.05, *⁣*^*∗∗*^*p*  < 0.01, *⁣*^*∗∗∗*^*p*  < 0.001, *⁣*^*∗∗∗∗*^*p*  < 0.0001.

Immune cell infiltration plays a crucial role in OA, highlighting its inflammatory and immune-related nature [26]. We applied the CIBERSORT algorithm to assess immune cell infiltration in OA and control samples. Comparative analysis revealed significant differences in immune cell composition between the two groups. Specifically, CD8^+^ T cells (*p*=0.0092) and regulatory T cells (Tregs, *p*=0.048) were significantly decreased in OA samples, while the proportions of activated NK cells (*p*=0.039), M1 macrophages (*p*=0.0026), resting mast cells (*p*=0.0025), and neutrophils (*p*=0.046) were notably increased (Figure 5A). In addition, we analyzed the correlation between oxidative stress signaling pathway scores and immune cell infiltration. The scores showed a positive correlation with M2 macrophages and activated NK cells, suggesting a potential link between oxidative stress and immune activation. Conversely, the scores were negatively correlated with resting mast cells and Tregs, indicating that elevated oxidative stress may be associated with reduced immunosuppressive activity and altered immune homeostasis in OA (Figure 5B). Correlation analysis between gene expression levels and immune cell infiltration revealed distinct immune regulatory roles of the key genes. COL6A1 expression was negatively correlated with CD8+ T cells (*r* = −0.353, *p*=0.014) and positively correlated with follicular helper T (Tfh) cells (*r* = 0.287, *p*=0.0048), suggesting its involvement in adaptive immune modulation. FOS showed a strong positive correlation with activated mast cells (*r* = 0.536, *p*=2.23e-08), implicating it in mast cell-mediated inflammatory responses. TP53 was negatively correlated with Tregs (*r* = −0.325, *p*=0.0013) but positively correlated with gamma delta T cells (*r* = 0.416, *p*=2.79e-05), indicating a potential role in shifting the immune balance toward proinflammatory and cytotoxic responses in OA (Figure 5C). Overall, oxidative stress and its related genes may impact the regression of OA by influencing the infiltration of immune cells.

### 3.6. Revealing the Biological Significance of FOS in OA via scRNA

**Figure 6:**
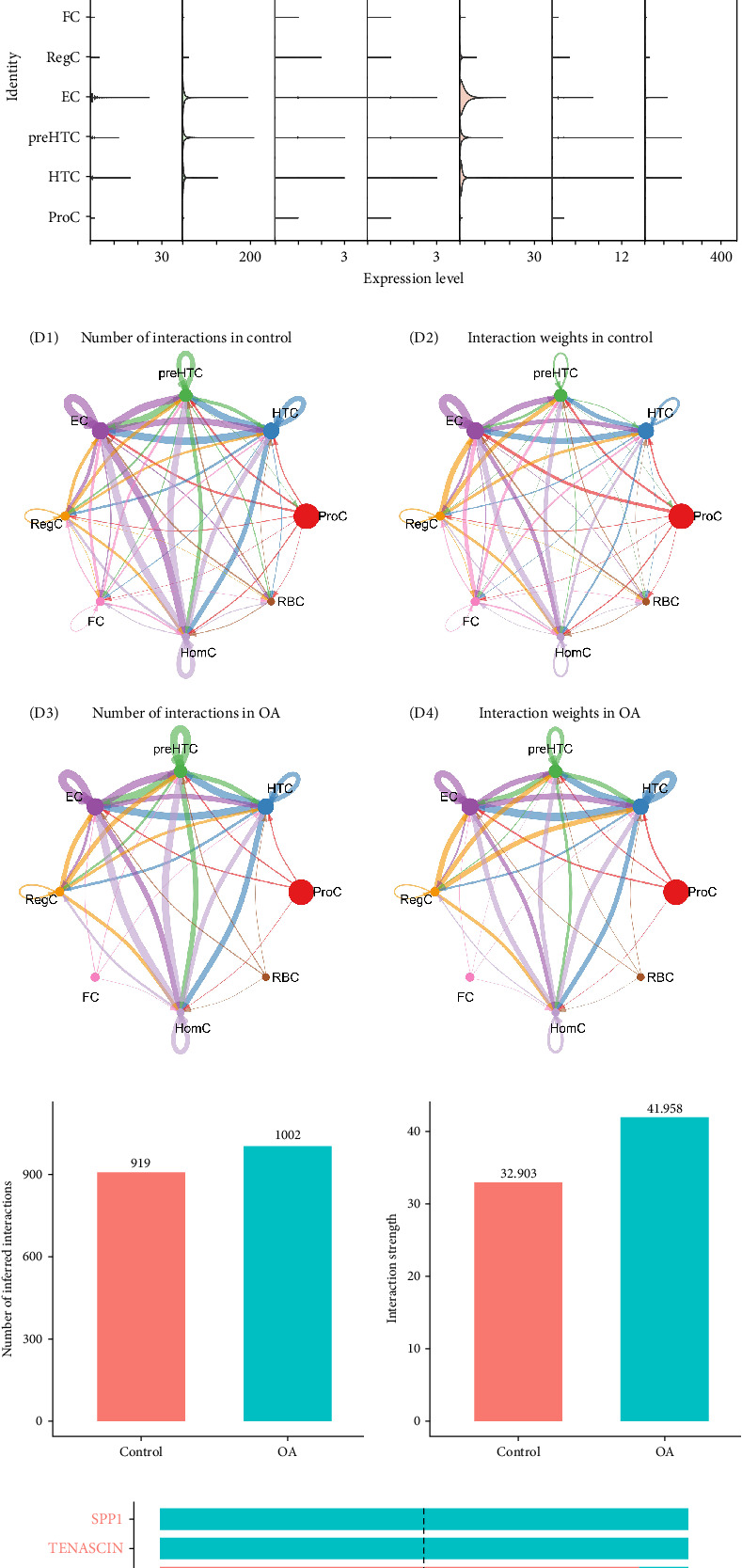
Uncovering the cellular localization of key gene expression and intercellular communication. (A) Comparison of key gene expression between OA and control samples. (B) The UMAP plot displays the expression levels of identified genes across cell types. (C) The violin plot displays the expression levels of identified genes across cell types. (D) Network of cell–cell communication among OA and control samples; number of interactions (D1, D3); strength of interactions (D2, D4). (E) Statistics of the number and strength of cell communication between OA and control samples. (F) Comparative analysis of signaling pathways between OA and control groups, with rankings denoting pathway significance. Red indicates pathways predominantly enriched in OA, green signifies those enriched in the control group, and black denotes pathways with no statistically significant enrichment differences. (G) Heatmap of cell–cell interactions among OA and control samples; number of interactions in control samples (G1); number of interactions in OA samples (G2). *⁣*^*∗∗*^*p* < 0.01, *⁣*^*∗∗∗∗*^*p* < 0.0001.

**Figure 7:**
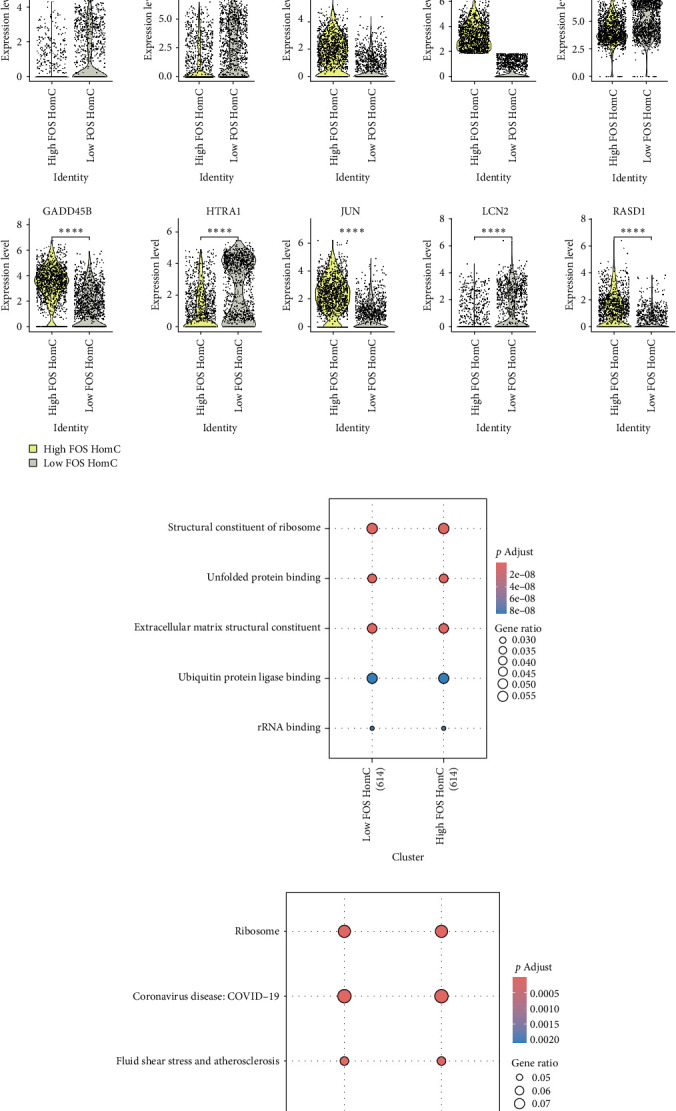
Analysis of the role of FOS in HomC cells at the single-cell RNA level. (A) Screening of key genes via Gosemsim. (B) The violin plots show the expression of FOS in different cell types between OA and control samples. (C) Comparison of the proportion of FOS-positive cells in OA and control samples. (D) The top 10 genes with the most significant differences between the high- and low-FOS HomC. (E, F) GO and KEGG enrichment analysis. (G) Identification of distinct alterations in Hallmark pathways between the high and low FOS expression HomC groups. *⁣*^*∗*^*p*  < 0.05, *⁣*^*∗∗*^*p*  < 0.01, *⁣*^*∗∗∗*^*p*  < 0.001, *⁣*^*∗∗∗∗*^*p*  < 0.0001.

As shown in Figure 6A, the expression characteristics of seven oxidative stress-related diagnostic genes (STC2, HSP1A, SELENON, TP53, COL6A1, LSP1, and FOS) were systematically analyzed at the single-cell level. Differential expression analysis revealed significant differences between OA and control samples, the expression trend of genes is consistent with the bulk transcriptome results. While UMAP and violin visualization further confirmed that these genes exhibited heterogeneous distribution patterns across distinct cell clusters, HSPA8, LSP1, and FOS are highly expressed in HomC, whereas COL6A1 is mainly localized in EC and HTC cell populations (Figure 6B,C), suggesting potential cell type–dependent functions in OA pathogenesis. To further explore the intercellular communication landscape, we reconstructed cell–cell interaction networks, which revealed extensive remodeling in OA compared with controls (Figure 6D). Quantitative comparison demonstrated that both the number and strength of cellular interactions were markedly increased in OA, indicative of intensified intercellular crosstalk (Figure 6E). Pathway-level analysis of relative information flow highlighted significant alterations in multiple signaling pathways, including extracellular matrix remodeling (e.g., collagen and integrin pathways) (Figure 6F). Further, the heatmap visualization provided additional evidence of signaling network reprogramming, showing pronounced differences in interaction strengths among immune cells, fibroblasts, and endothelial populations in OA (Figure 6G). We subsequently used the GOSemSim method to evaluate the functional similarity among the seven key genes based on GO terms, allowing us to rank their relative importance [27]. The analysis revealed that FOS had the highest semantic similarity scores with the other genes (Figure 7A), suggesting that it shares the most common biological functions and pathways. This highlights FOS as a potential hub gene, likely playing a central regulatory role in the molecular mechanisms underlying OA, and underscores its value as a promising target for further functional investigation and therapeutic intervention. We further analyzed the expression differences of FOS across single-cell subsets and found that FOS expression was significantly elevated in HomC from OA samples compared to controls (Figure 7B). This finding suggests a potential cell-type-specific role of FOS in OA pathology. To investigate the potential biological impact of this upregulation, we stratified HomC cells into high- and low-FOS expression groups. Notably, the proportion of HomC cells with high FOS expression was markedly higher in OA (74%) than in the control group (49%) (Figure 7C). The top 10 genes showing the most significant differential expression between the high- and low-FOS expression groups were C15orf48, CCL20, EGR1, FOS, FTH1, GADD45B, HTRA1, JUN, LCN2, and RASD1. Among these, EGR1, FOS, GADD45B, JUN, and RASD1 were markedly upregulated in the FOS-high group (Figure 7D). In addition, we performed GO and KEGG enrichment analyses based on the DEGs between the high- and low-FOS expression groups. GO analysis revealed significant enrichment in pathways such as the structural constituent of ribosome, ECM structural constituent, and ubiquitin protein ligase binding (Figure 7E), suggesting involvement in protein synthesis, matrix remodeling, and protein degradation. KEGG pathway analysis highlighted enrichment in spliceosome and osteoclast differentiation (Figure 7F), indicating that alternative splicing regulation and bone-resorptive processes may be influenced by FOS activity in OA-related HomC cells. A PPI network was constructed based on the top 10 DEGs between FOS-high and FOS-low HomCs to evaluate whether FOS interacts with other genes (Supporting Information 3: Figure S2A). Notably, FOS exhibited the highest number of interaction nodes within the network validated by the Degree method embedded in the cytoHubba plugin (Supporting Information 3: Figure S2B), suggesting its central role as a hub gene in regulating the associated transcriptional program. Furthermore, we applied the irGSEA algorithm to assess HALLMARK pathway activity based on gene sets curated from the MsigDB database, which enabled us to profile functional differences between FOS-high and FOS-low HomC cells at the pathway level. The analysis revealed that the INFLAMMATORY_RESPONSE pathway was predominantly upregulated in HomC cells with low FOS expression, whereas pathways, including TNFA_SIGNALING_VIA_NFKB, TGF_BETA_SIGNALING, P53_PATHWAY, and G2M_CHECKPOINT were notably elevated in the high-FOS HomC population (Figure 7G). We employed the CellChat package to systematically analyze the intercellular communication networks involving HomC and other cell types. The results demonstrated that the ligand–receptor interaction patterns of HomC differed significantly between the OA and control groups, suggesting disease-specific alterations in cellular signaling dynamics (Supporting Information 3: Figure S2). In the OA group, HomC cells were predicted to interact with HTC, preHTC, EC, and other cell types primarily through ligand-receptor pairs such as FN1–CD44, FN1–SDC4, and COMP–SDC4. In contrast, in the normal control group, HomC communication appeared to be mediated mainly via the COL9A3–CD44 axis, indicating distinct cell–cell interaction profiles associated with the disease state (Supporting Information 4: Figure S3).

### 3.7. Screening and Verifying Potential Targeted Drugs

**Figure 8:**
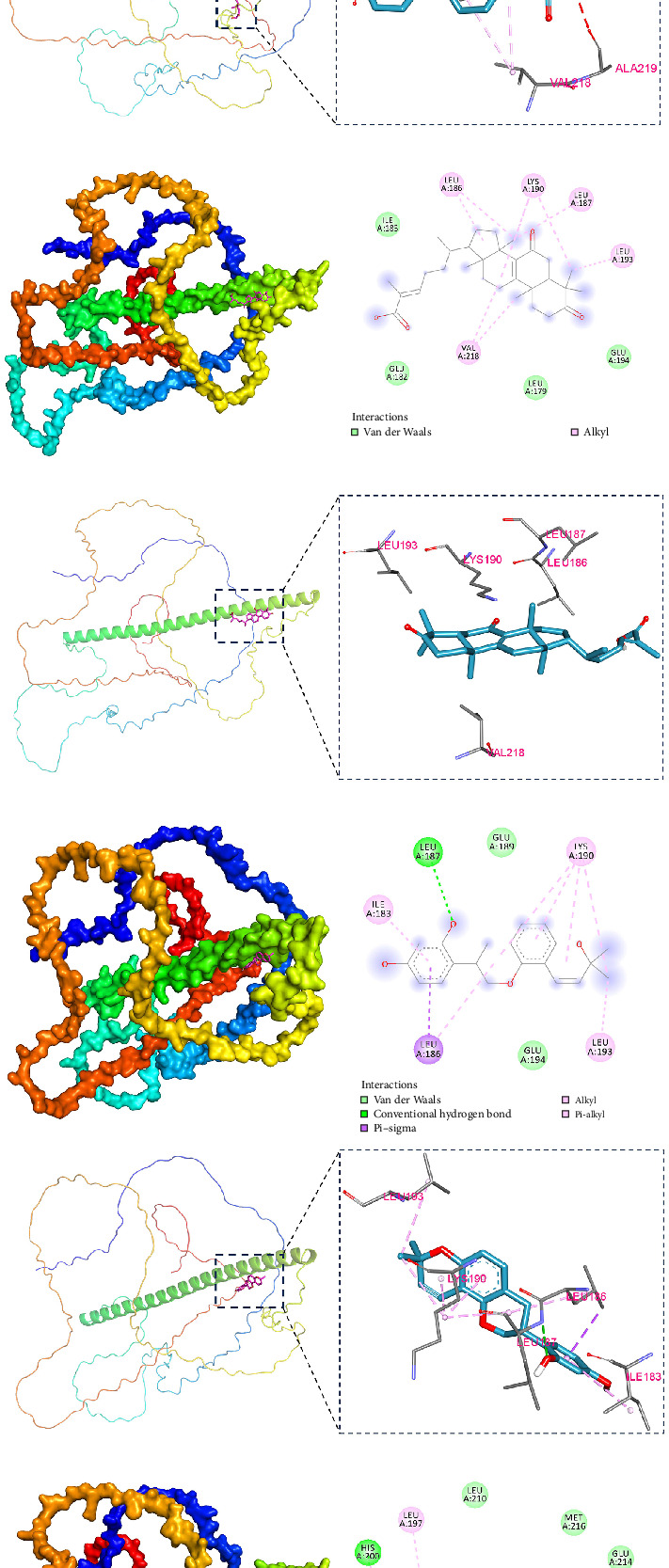
Prediction network and molecular docking of potential drugs targeting FOS. (A) Eighteen potential small-molecule drugs targeting FOS were identified using the BATMAN database. (B–F) The top five significant molecular docking diagrams: (B) FOS-18alpha-glycyrrhetinic acid (−7.364 kcal/mol), (C) FOS-desoxycortone (−6.412 kcal/mol), (D) FOS-ganoderic acid DM (−6.674 kcal/mol), (E) FOS-glabridin (−6.674 kcal/mol), and (F) FOS-ursolic acid (−7.369 kcal/mol). (G) RMSD values of the protein–ligand complex over time, (H) Rg values of the protein–ligand complex over time, (I) SASA values of the protein–ligand complex over time, (J) H-bonds values of the protein–ligand complex over time, (K) RMSF values of the protein–ligand complex.

We further identified 18 small-molecule compounds predicted to interact with the FOS protein and conducted molecular docking analyses to assess their binding affinities and interaction profiles (Figure 8A). Among these, the top five compounds with the highest predicted binding affinities were 18α-glycyrrhetinic acid (−7.364 kcal/mol), desoxycortone (−6.412 kcal/mol), ganoderic acid DM (−6.674 kcal/mol), glabridin (−6.674 kcal/mol), and UA (−7.369 kcal/mol), detailed structural visualization of the docking poses, including key binding residues and interaction sites, is provided in Figure 8B–F. 18α-glycyrrhetinic acid forms hydrogen bonds with the LEU197 residue of FOS (Figure 8B). Desoxycortone interacts with LEU186, LYS190, and VAL218 through hydrophobic interactions (Figure 8C). Ganoderic acid DM engages in hydrophobic interactions with LEU186, LEU193, LYS190, and VAL218 (Figure 8D). Glabridin binds to LEU186 via Pi–Sigma interactions, forms hydrogen bonds with LEU187, and interacts hydrophobically with ILE183, LYS190, and LEU193 (Figure 8E). UA, the small molecule exhibiting the highest binding affinity, primarily interacts with FOS through conventional hydrogen bonds with HIS200 and hydrophobic interactions with LEU197, CYS204, and ARG201 (Figure 8F). These compounds demonstrated strong and stable docking conformations with FOS, suggesting their potential as candidate inhibitors or modulators. To evaluate the binding stability of small molecule UA - the highest binding affinity - with FOS, molecular dynamics simulations were performed. It demonstrated that the UA–FOS complex maintained structural stability throughout the 100 ns trajectory. The RMSD reached equilibrium after ~ 80 ns with fluctuations around 3.9 Å, while Rg and SASA values showed only minor variations, indicating limited conformational changes (Figure 8). The complex maintained an average of ~4 hydrogen bonds during the simulation, supporting favorable ligand–protein interactions (Figure 8J). RMSF values remained low (<3 Å), further confirming reduced flexibility and high stability of the complex (Figures 8K and 7K). Collectively, these results suggest that UA binds stably to FOS with strong hydrogen-bonding interactions.

### 3.8. FOS Knockdown and UA Treatment Attenuate IL-1β-Induced Oxidative Stress, Apoptosis, and Inflammation in Chondrocytes In Vitro

**Figure 9:**
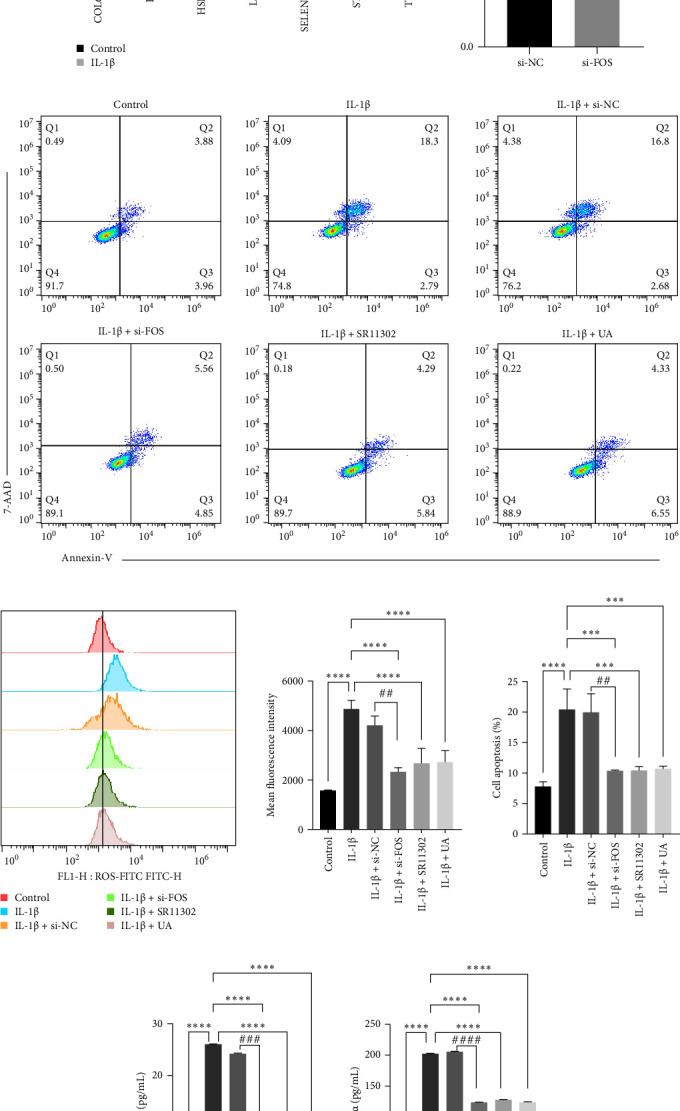
FOS knockdown attenuates IL-1β-induced ROS production, apoptosis, and proinflammatory cytokine release in IL-1β-treated SW1353 cell line. (A) qRT-PCR analysis of oxidative stress–related genes in control and IL-1β-treated chondrocytes. (B) Validation of FOS knockdown efficiency by siRNA. (C) Annexin V/7-AAD flow cytometry showing that si-FOS, SR11302, or UA treatment reduced IL-1β-induced apoptosis compared with IL-1β + si-NC. (D) Flow cytometry analysis of ROS levels using DCFH-DA staining. (E) ELISA quantification of IL-6 and TNF-α secretion. *⁣*^*∗*^*p*  < 0.05, *⁣*^*∗∗*^*p*  < 0.01, *⁣*^*∗∗∗*^*p*  < 0.001, *⁣*^*∗∗∗∗*^*p*  < 0.0001; *⁣*^##^*p*  < 0.01, *⁣*^###^*p*  < 0.001, IL-1β + si-FOS vs. IL-1β + si-NC.

In the IL-1β stimulation model, qRT-PCR analysis demonstrated that all oxidative stress–related genes identified in our model, including STC2, LSP1, COL6A1, FOS, SELENON, TP53, and HSPA8, exhibited significant transcriptional changes compared with the control group (Figure 9A, all *p* < 0.05). To clarify the role of FOS in IL-1β-induced oxidative stress and inflammation in chondrocytes, we next performed targeted knockdown of FOS using siRNA to examine its functional involvement in the SW1353 cell line. Transfection with si-FOS significantly reduced FOS mRNA expression compared with negative control siRNA (si-NC) (Figure 9B, *p* < 0.001), confirming the efficiency of gene silencing. To further benchmark the effect of genetic inhibition, we included SR11302, a well-characterized pharmacological inhibitor of FOS [28], as a positive control, and UA, a natural compound predicted to target FOS, as a candidate therapeutic agent. Flow cytometric analysis of annexin V/7-AAD staining demonstrated that IL-1β stimulation led to a marked increase in the proportion of apoptotic chondrocytes compared with the control condition (20.44% ± 3.368% vs. 7.84% ± 0.76%, *p*  < 0.0001, Figure 9C). Compared with the negative control group, FOS knockdown (10.41% ± 0.155%) significantly reduced cell apoptosis (10.41% ± 0.155% vs. 19.97% ± 3.07%, *p*  < 0.001), producing an effect comparable in magnitude to that of SR11302 treatment (10.44% ± 0.653%). Notably, UA administration (10.73% ± 0.441%) also reduced apoptosis to a similar extent, indicating that it may mimic the inhibitory action of FOS silencing. In parallel, intracellular ROS levels were assessed by DCFH-DA–based flow cytometry. IL-1β stimulation caused a substantial elevation in mean fluorescence intensity, indicating excessive ROS production (*p*  < 0.0001, Figure 9D). Both si-FOS and SR11302 markedly suppressed ROS accumulation, while UA treatment yielded a comparable decrease, further supporting its potential role as a functional FOS inhibitor. To determine whether FOS modulation influences inflammatory mediator release, we measured IL-6 and TNF-α levels in culture supernatants using ELISA. Consistent with the oxidative stress and apoptosis results, IL-1β markedly increased IL-6 (3.47 ± 0.118 pg/mL vs. 26.03 ± 0.097 pg/mL, *p*  < 0.0001) and TNF-α (38.06 ± 0.203 pg/mL vs. 202.7 ± 0.322 pg/mL, *p*  < 0.0001) secretion compared with the control group (Figure 9E). Both genetic silencing of FOS (IL6: 9.162 ± 0.054 pg/mL, TNF-α: 124.0 ± 0.300 pg/mL) and pharmacological inhibition with SR11302 (IL6: 8.466 ± 0.541 pg/mL, TNF-α: 128.3 ± 0.231 pg/mL) significantly reduced these cytokine levels. Importantly, UA administration also produced a substantial decrease in IL-6 (8.549 ± 0.426 pg/mL) and TNF-α (124.0 ± 0.153 pg/mL) secretion, demonstrating that its anti-inflammatory effect parallels that of direct FOS inhibition. Collectively, these findings indicate that FOS is a critical mediator of IL-1β-induced oxidative stress, apoptosis, and inflammatory responses in chondrocytes. Furthermore, it suggests that pharmacological targeting of FOS, particularly with UA, may represent a promising therapeutic strategy for mitigating chondrocyte injury in inflammatory joint diseases.

## 4. Discussion

In this study, we systematically investigated the involvement of oxidative stress signaling pathways in OA by integrating bulk RNA sequencing and single-cell transcriptomic data. The GSEA result of the GSE236924 dataset revealed significant enrichment of oxidative stress-related gene signatures in OA tissues, consistent with previous findings that oxidative stress plays a central role in the pathogenesis of OA [29, 30]. Elevated oxidative stress pathway scores in OA tissues further support the notion that oxidative damage may contribute to cartilage degradation and synovial inflammation. Differential expression analysis identified 58 oxidative stress-related genes with significant alterations in OA samples, including genes involved in redox homeostasis and antioxidant responses. GO enrichment analysis confirmed their association with BPs such as response to oxidative stress, antioxidant activity, and peroxidase function. These findings are consistent with established literature indicating that oxidative stress impairs chondrocyte function and matrix synthesis, thereby accelerating OA progression [31]. Furthermore, KEGG pathway analysis indicated the enrichment of genes in the longevity-regulating pathway and inflammatory signaling pathways such as TNF, PI3K-Akt, and IL-17 pathways [10, 32, 33]. It reflects the interplay between oxidative stress and chronic inflammation in OA, as ROS generation can activate proinflammatory cascades that exacerbate joint damage [9]. We further leveraged the high-resolution power of scRNA-seq to delineate the distribution of oxidative stress signaling across distinct chondrocyte subpopulations. By using the Harmony algorithm for strict quality control and batch effect correction, we identified nine major cell clusters and annotated them according to typical marker genes, and the cell clustering results were similar to those of Ji et al. [20]. Among these, HomCs displayed the highest oxidative stress signaling scores, underscoring their potential central role in maintaining intracellular redox homeostasis. HomCs displayed the highest oxidative stress activity, likely due to their high expression of metallothionein genes, which are essential for regulating intracellular ROS and maintaining cartilage homeostasis [34]. In OA, however, HomCs were markedly reduced and exhibited a shift in gene expression from homeostatic pathways (protein processing, immune regulation, and cellular maintenance) toward pathological processes, including ECM degradation and ossification [35]. These findings suggest that excessive oxidative stress may disrupt the protective role of HomCs, contributing to their dysfunction and the progression of OA. Conversely, ProCs and FCs exhibited relatively lower scores, suggesting differential vulnerability or adaptive responses to oxidative stress among chondrocyte subtypes. Interestingly, oxidative stress signaling scores were significantly elevated in several OA-specific chondrocyte subtypes, including HTCs, ECs, and RegCs, implying that oxidative stress may drive pathological phenotypic transitions in OA. To our knowledge, this is the first study to systematically characterize the oxidative stress signaling landscape across chondrocyte subpopulations at single-cell resolution. This cell-type-specific variation highlights the heterogeneous nature of oxidative stress responses and underscores the importance of tailored therapeutic strategies targeting redox imbalance in specific chondrocyte subsets. Collectively, our findings corroborate previous studies implicating oxidative stress as a key pathological driver in OA and further emphasize its heterogeneous distribution among chondrocyte populations at the single-cell level. These insights provide a refined understanding of oxidative stress dynamics in OA and may guide the development of cell-targeted antioxidant therapies.

We identified seven oxidative stress-related genes, STC2, LSP1, COL6A1, FOS, SELENON, TP53, and HSPA8, as potential diagnostic biomarkers for OA using LASSO regression. These genes were selected through LASSO regression analysis and demonstrated robust diagnostic performance in both training and validation cohorts, as evidenced by high AUC values in ROC curve analysis. Notably, STC2 was the only gene downregulated in OA tissues, suggesting a potentially protective role, whereas the others were upregulated, indicating their possible involvement in OA pathogenesis. Among the seven genes, STC2 was uniquely downregulated in OA tissues. STC2 is known to play a protective role under oxidative stress by enhancing cell survival through the suppression of ROS and modulation of ER stress [36, 37]. Its downregulation may impair the oxidative defense mechanisms in chondrocytes, potentially contributing to cellular senescence and ECM degradation in OA. Interestingly, STC2 was also found to be negatively correlated with hallmark inflammatory and metabolic pathways, including NOTCH signaling and nitrogen metabolism, suggesting a potential role in attenuating inflammation within the osteoarthritic microenvironment. Notably, a study by Robert H. et al. demonstrated that intravitreal administration of Stanniocalcin-1 effectively attenuated oxidative stress and inflammation in a retinitis pigmentosa model [38], highlighting its anti-inflammatory potential. These findings provide a conceptual basis for exploring intra-articular injection of STC2 to enhance its local expression and modulate the oxidative and inflammatory milieu in OA. Conversely, the remaining six genes, LSP1, COL6A1, FOS, SELENON, TP53, and HSPA8, were upregulated in OA tissues, indicating their potential roles as risk factors. Notably, TP53, a classical tumor suppressor gene, is implicated in chondrocyte apoptosis and senescence in OA [39]. Its increased expression may reflect an attempt to eliminate damaged cells in the OA joint, although sustained activation could exacerbate tissue degeneration. SELENON, a selenium-containing antioxidant enzyme, is known to modulate redox balance through the Nrf2 pathway [40]. Its upregulation may represent a compensatory response to oxidative injury in OA, particularly in synovial tissues. Additionally, HSPA8, a molecular chaperone, functions as a molecular chaperone that assists in the proper folding of nascent and stress-denatured proteins. Importantly, it plays a central role in chaperone-mediated autophagy (CMA), a selective autophagic process crucial for removing oxidized or dysfunctional proteins. Impaired autophagy is a hallmark of OA and contributes to chondrocyte senescence and apoptosis [41]. HSPA8 was also found to be a potentially effective diagnostic marker for OA in another autophagy-centered study [42]. HSPA8 was enriched in the TCA cycle and amino sugar metabolism pathways, indicating its involvement in energy homeostasis and stress protein regulation within degenerating cartilage. The positive correlation of HSPA8 and TP53 with hallmark pathways such as APOPTOSIS and INFLAMMATORY_RESPONSE supports their potential synergistic role in chondrocyte loss and inflammation. Interestingly, COL6A1 and LSP1 were significantly enriched in ECM-related and immune-regulatory pathways. COL6A1, a critical ECM component, was associated with glycosaminoglycan metabolism and fatty acid metabolism, suggesting dual roles in structural support and metabolic adaptation in OA cartilage [43]. LSP1, typically involved in leukocyte migration and inflammation, showed correlations with both glycan biosynthesis and the renin–angiotensin system, potentially linking immune infiltration with vascular remodeling in the OA joint [44].

Pathway analysis further highlighted the complexity of OA pathogenesis. Hallmark pathways such as inflammatory response, epithelial-mesenchymal transition (EMT), and NOTCH signaling were upregulated in OA samples, reflecting a convergence of proliferative, inflammatory, and fibrotic processes that underlie joint degeneration [45–47]. Notably, STC2 was negatively correlated with NOTCH signaling, which has been implicated in chondrocyte hypertrophy and matrix breakdown [47], suggesting a protective, regulatory function that may counteract OA progression. Our GSEA findings also revealed a marked downregulation of fatty acid metabolism, myogenesis, and hypoxia-related pathways, which may indicate metabolic inflexibility and impaired adaptation to hypoxic stress in OA cartilage. The enrichment of genes like COL6A1, HSPA8, and SELENON in metabolic pathways points to a metabolic reprograming that could reflect both adaptive and maladaptive responses to chronic joint stress. The correlation matrix and pathway enrichment patterns also suggest that these key genes do not act in isolation but likely participate in an intricate regulatory network. For example, LSP1 was positively correlated with HSPA8 but negatively with SELENON and TP53, hinting at potential antagonistic or context-dependent gene–gene interactions. This complexity supports the notion of OA as a heterogeneous disease with possible molecular subtypes, where differential pathway activity may underlie variability in clinical outcomes and therapeutic responses. In summary, our integrated analysis underscores the multifactorial nature of OA, implicating oxidative stress-related genes in diverse BPs such as inflammation, apoptosis, ECM remodeling, and metabolism. These seven genes hold promise as diagnostic biomarkers and may serve as potential therapeutic targets. Future studies leveraging spatial transcriptomics and multiomics integration may further elucidate their spatiotemporal roles in OA pathogenesis and progression.

Unlike the earlier view of OA as a purely degenerative joint disorder driven by mechanical wear and cartilage erosion, emerging evidence highlights the active involvement of both innate and adaptive immune responses in the synovial membrane, subchondral bone, and cartilage [48]. The accumulation of immune cells, including macrophages, T lymphocytes, B cells, and mast cells, within the joint microenvironment contributes to the sustained production of proinflammatory cytokines, chemokines, and matrix-degrading enzymes, thereby accelerating cartilage destruction, synovitis, and osteophyte formation [49]. This immunological perspective of OA provides a more comprehensive framework for understanding its pathophysiology and opens new avenues for the development of targeted immunomodulatory therapies. In this study, we employed the CIBERSORT algorithm to systematically quantify immune cell populations in OA and control synovial tissues, revealing significant alterations in the immune landscape associated with OA pathogenesis. Notably, we observed a marked decrease in CD8^+^ T cells and Tregs in OA samples, accompanied by increased infiltration of activated NK cells, M1 macrophages, resting mast cells, and neutrophils. These shifts reflect a transition toward a proinflammatory immune milieu, potentially amplifying cartilage degradation and synovial inflammation. The reduction in Tregs, key mediators of immune tolerance, suggests impaired immunosuppressive mechanisms in OA. Previous studies have confirmed the protective role of Tregs in limiting joint inflammation and preserving cartilage integrity [50]. Their depletion in OA may contribute to unchecked inflammatory responses and synovial hyperplasia. Similarly, reduced CD8^+^ T cells, which can modulate inflammation and cytotoxicity, align with prior observations of altered adaptive immune responses in degenerative joint diseases [51]. On the other hand, the enrichment of M1 macrophages, which are known for their proinflammatory phenotype, supports existing data implicating these cells in synovial inflammation, cytokine production (e.g., IL-1β, TNF-α), and matrix metalloproteinase activation in OA [52]. Interestingly, the oxidative stress signaling pathway scores were positively correlated with M2 macrophages and activated NK cells, while negatively correlated with resting mast cells and Tregs. These findings provide compelling evidence for a bidirectional crosstalk between oxidative stress and immune dysregulation in OA. Oxidative stress, driven by an imbalance between ROS production and antioxidant defenses, has been shown to activate immune responses and modulate immune cell recruitment [9]. The observed inverse relationship with Tregs suggests that elevated oxidative stress may suppress immunoregulatory pathways, favoring chronic inflammation [53]. Interestingly, the oxidative stress signaling pathway scores were positively correlated with M2 macrophages and activated NK cells, while negatively correlated with resting mast cells and Tregs. These findings provide compelling evidence for a bidirectional crosstalk between oxidative stress and immune dysregulation in OA. Oxidative stress, driven by an imbalance between ROS production and antioxidant defenses, has been shown to activate immune responses and modulate immune cell recruitment [54]. The observed inverse relationship with Tregs suggests that elevated oxidative stress may suppress immunoregulatory pathways, favoring chronic inflammation. Moreover, gene-immune correlation analysis highlighted distinct immunomodulatory roles of key oxidative stress-related genes. For example, COL6A1 exhibited a negative correlation with CD8^+^ T cells and a positive correlation with Tfh cells, implicating this ECM protein in shaping adaptive immunity. These findings align with reports linking COL6A1 to matrix remodeling and its interaction with the immune cells of macrophages [43]. The strong positive correlation between FOS expression and activated mast cells suggests that FOS may facilitate mast cell-driven inflammatory responses, consistent with its established role as a transcription factor downstream of MAPK and cytokine signaling cascades [55]. Collectively, our findings support the hypothesis that oxidative stress and its associated gene network modulate OA progression through the regulation of immune cell infiltration and immune signaling pathways. The interplay between redox imbalance and immune dysregulation may not only exacerbate joint inflammation and cartilage damage but also represent a critical therapeutic target for OA management. Future studies should further dissect the mechanistic pathways linking oxidative stress with immune cell phenotypes and explore the potential of redox-immune biomarkers for OA diagnosis and treatment stratification.

FOS, a key component of the activator protein-1 (AP-1) transcription factor complex, plays a central role in regulating cellular processes such as proliferation, differentiation, apoptosis, and inflammation [56]. As an immediate early response gene, FOS is rapidly induced by various stimuli, including oxidative stress, proinflammatory cytokines, and mechanical injury, all of which are critical pathological factors in OA [57, 58]. In this study, we leveraged GO semantic similarity analysis to assess the functional relationships among seven oxidative stress-related genes implicated in OA. Among them, FOS emerged as the gene with the highest semantic similarity to the others, suggesting its involvement in a wide array of shared BPs and positioning it as a likely hub gene in the OA gene regulatory network. Knockdown of FOS expression has been shown to enhance cell proliferation and promote cartilage anabolism, while simultaneously suppressing cellular senescence and inhibiting cartilage catabolic processes [59]. By downregulating FOS, the balance within cartilage tissue shifts toward matrix synthesis and repair, supporting chondrocyte viability and function. Additionally, reduced FOS activity mitigates the expression of catabolic enzymes, such as MMPs, and decreases inflammatory signaling, thereby preserving ECM integrity and potentially slowing the progression of OA [60]. However, FOS-mediated metabolic reprograming is involved in the pathogenesis of OA that regulates cellular bioenergetics and maintains cartilage integrity via the TCA cycle/oxidative phosphorylation (OXPHOS) [61]. This contradictory evidence prompted us to further explore the role of FOS in OA. Single-cell analysis further revealed that FOS expression was significantly elevated in HomC cells, a subset likely corresponding to homeostatic or cartilage-resident chondrocytes, in OA compared to controls. The decrease of HomCs in OA cartilage impaired matrix synthesis ability, and the increased inflammatory level led to the progression of OA, which may be related to the elevated FOS. This cell-type-specific upregulation indicates that FOS may exert key functional effects in OA at the cellular level. Stratification of HomC cells based on FOS expression revealed not only a higher proportion of FOS-high cells in OA joints but also distinct transcriptional profiles characterized by the upregulation of inflammation- and stress-related genes such as JUN, EGR1, GADD45B, and RASD1. Interestingly, hallmark pathway analysis using irGSEA revealed divergent functional states between FOS-high and FOS-low HomC cells. While FOS-low cells exhibited elevated inflammatory response signatures, FOS-high cells were enriched for TNF-α signaling via NF-κB, p53 pathway, TGF-β signaling, and G2M checkpoint pathways. These findings suggest that high FOS expression may be associated with cellular stress responses, proliferation, and tissue remodeling, while lower FOS expression is linked to acute inflammatory activity. Although the data from this study and external data highlight FOS as a central molecular player in OA, further experiments are still needed to verify its specific role in OA.

## Data Availability

The datasets from the public databases are available through the website of the paper. If other data are required, please contact the corresponding author if there are justifiable reasons.

## Supporting Information

Supporting Information 1 Table S1: Sequencing of homo gene primers.

Supporting Information 2 Figure S1: Correlation of key genes with Hallmark pathway scores. (A) Differences in Hallmark pathway scores between disease and control groups. (B) Analysis of the correlation between key genes and Hallmark pathway scores. Red represents positive correlation, with deeper shades indicating stronger positive correlation. Green represents negative correlation, with deeper shades indicating stronger negative correlation. *⁣*^*∗*^*p* < 0.05, *⁣*^*∗∗*^*p* < 0.01, *⁣*^*∗∗∗*^*p* < 0.001, *⁣*^*∗∗∗∗*^*p* < 0.0001.

Supporting Information 3 Figure S2: PPI analysis based on the top 10 DEGs between FOS-high and FOS-low HomCs. (A) PPI network and (B) top 10 genes in the network ranked by the Degree method.

Supporting Information 4 Figure S3: Intercellular communication signals between HomC and other cell types in OA and normal tissues. The darker the color, the greater the probability of communication between cells.
