# Supplementary material for: Integrative Bulk and Single-Cell Transcriptomic Profiling Reveals Oxidative Stress-Related Genes and Potential Therapeutic Targets in Osteoarthritis
Source: Mediators Inflamm. 2025 Oct 10;2025:1240226. doi: 10.1155/mi/1240226 (PMC12534163; doi:10.1155/mi/1240226)
Supplement: Supporting Information 1 — Table S1: Sequencing of homo gene primers. [file 1240226.f1.docx]

**Table S1.** **Sequencing of homo gene primers applied in the Q-PCR.**

| **Gene** | **Forward Sequence (5' -> 3')** | **Reverse Sequence (5' -> 3')** |
| --- | --- | --- |
| **STC2** | GCGTGCAGGTTCAGTGTGA | GGCCAGTCTCCCTACTGCT |
| **LSP1** | TCCGTCCTGCAAGGATATTGT | ATGGGGTGCTCTTAATTGTTGAT |
| **COL6A1** | AGGGCTACAAGGAACCCTGT | CACCGAGAAGACTTTGACGC |
| **FOS** | CCGGGGATAGCCTCTCTTACT | CCAGGTCCGTGCAGAAGTC |
| **SELENON** | CCTGAGCATGTTCCACCCTC | GATGTGGCCGGTGAACTGA |
| **TP53** | GAGGTTGGCTCTGACTGTACC | TCCGTCCCAGTAGATTACCAC |
| **HSPA8** | ACCTACTCTTGTGTGGGTGTT | GACATAGCTTGGAGTGGTTCG |
