# Supplementary figures and images for: Integrative Bulk and Single-Cell Transcriptomic Profiling Reveals Oxidative Stress-Related Genes and Potential Therapeutic Targets in Osteoarthritis
Source: Mediators Inflamm. 2025 Oct 10;2025:1240226. doi: 10.1155/mi/1240226 (PMC12534163; doi:10.1155/mi/1240226)

A

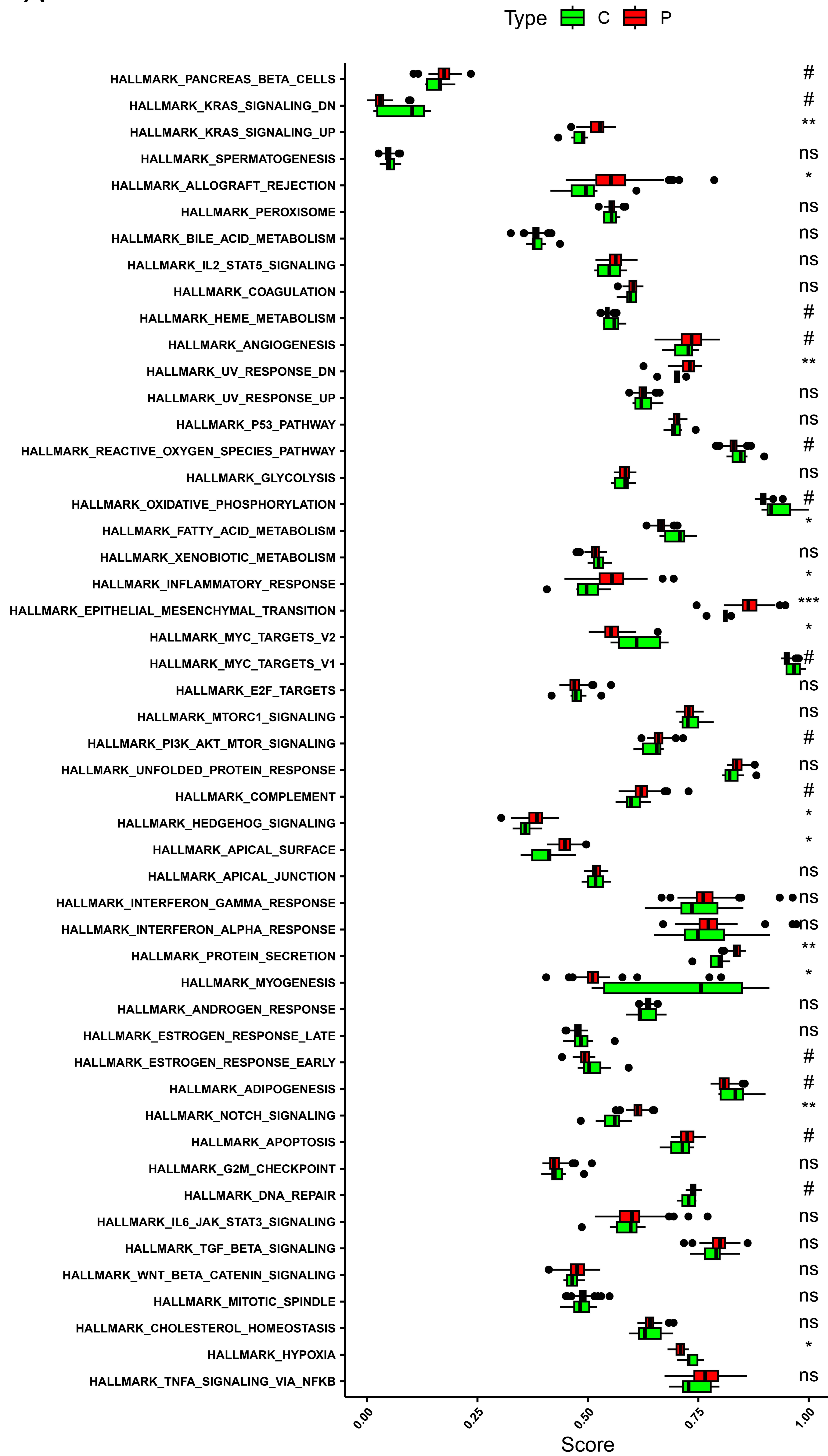

B

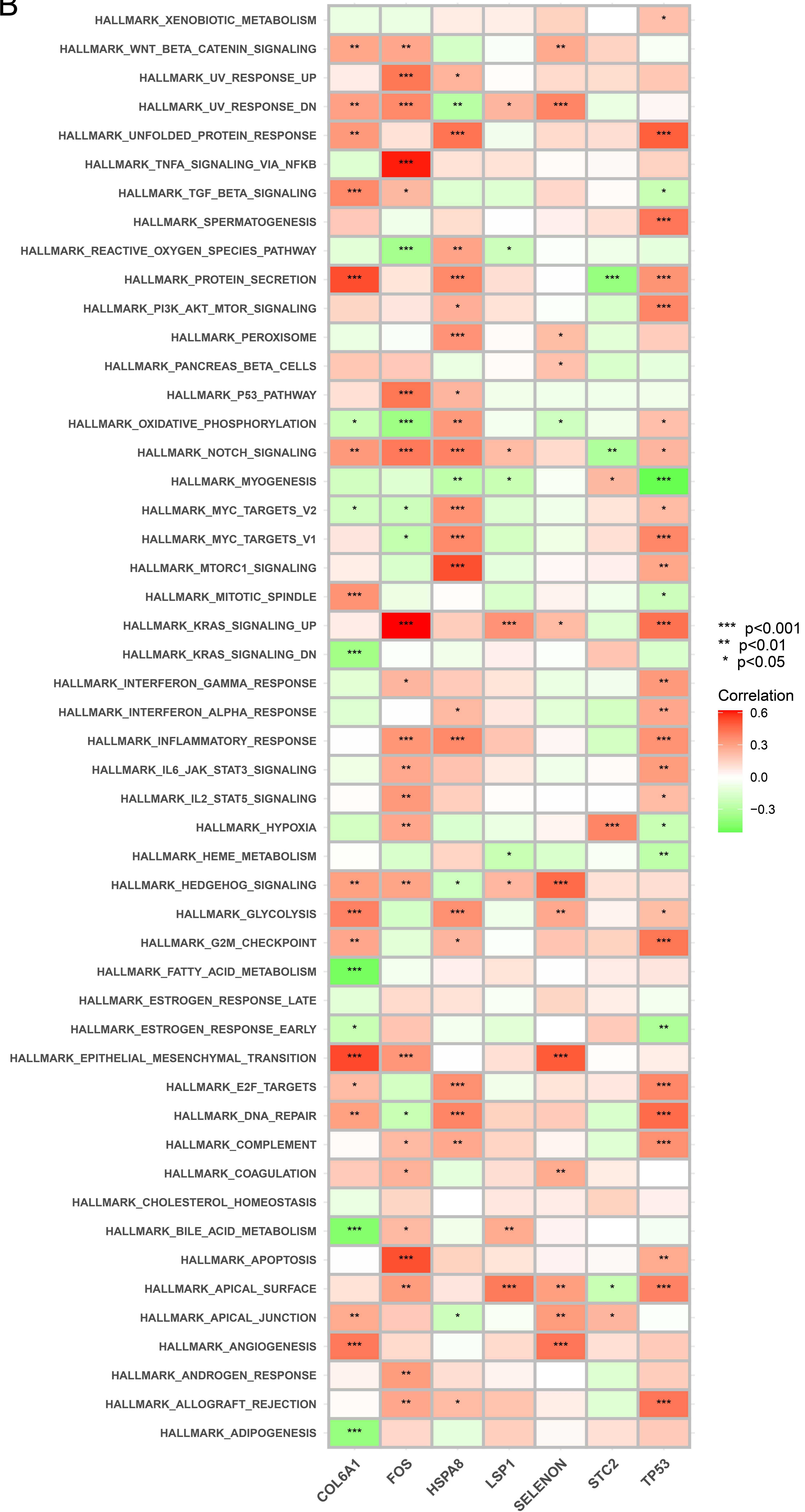

Supplement: Supporting Information 2 — Figure S1: Correlation of key genes with Hallmark pathway scores. (A) Differences in Hallmark pathway scores between disease and control groups. (B) Analysis of the correlation between key genes and Hallmark pathway scores. Red represents positive correlation, with deeper shades indicating stronger positive correlation. Green represents negative correlation, with deeper shades indicating stronger negative correlation. ⁣∗p < 0.05, ⁣∗∗p < 0.01, ⁣∗∗∗p < 0.001, ⁣∗∗∗∗p < 0.0001. [file 1240226.f2.pdf]

A

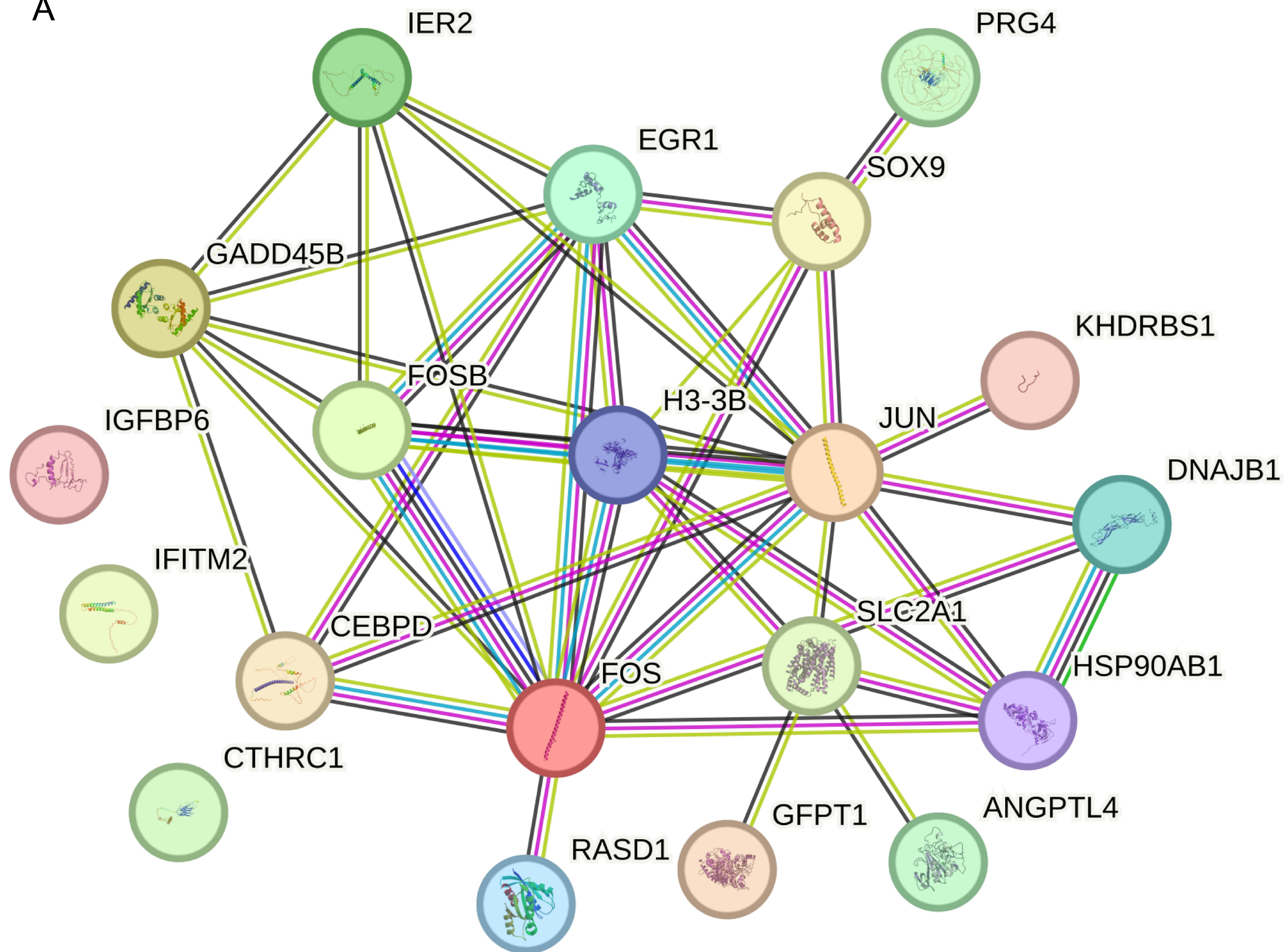

B

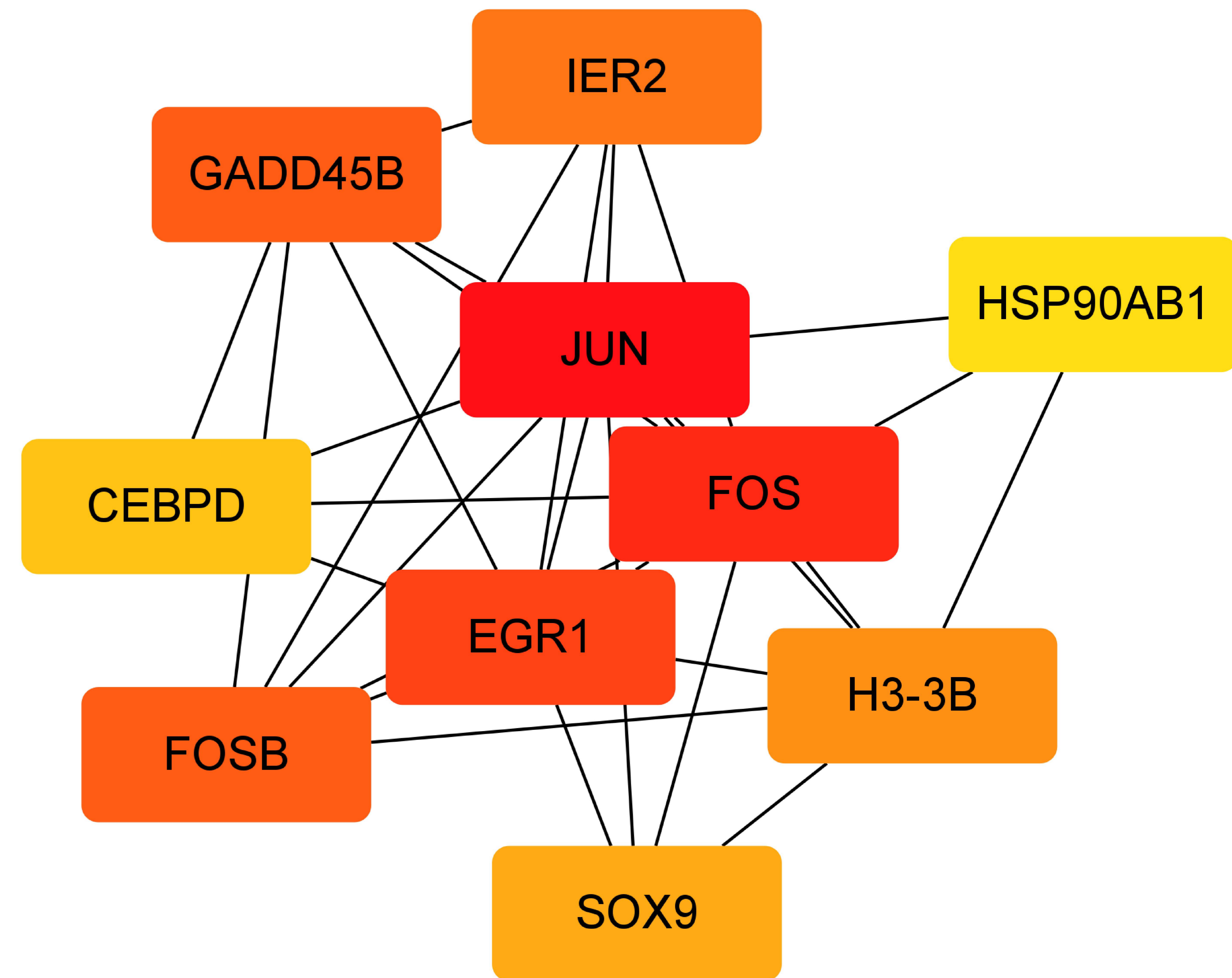

Supplement: Supporting Information 3 — Figure S2: PPI analysis based on the top 10 DEGs between FOS-high and FOS-low HomCs. (A) PPI network and (B) top 10 genes in the network ranked by the Degree method. [file 1240226.f3.pdf]

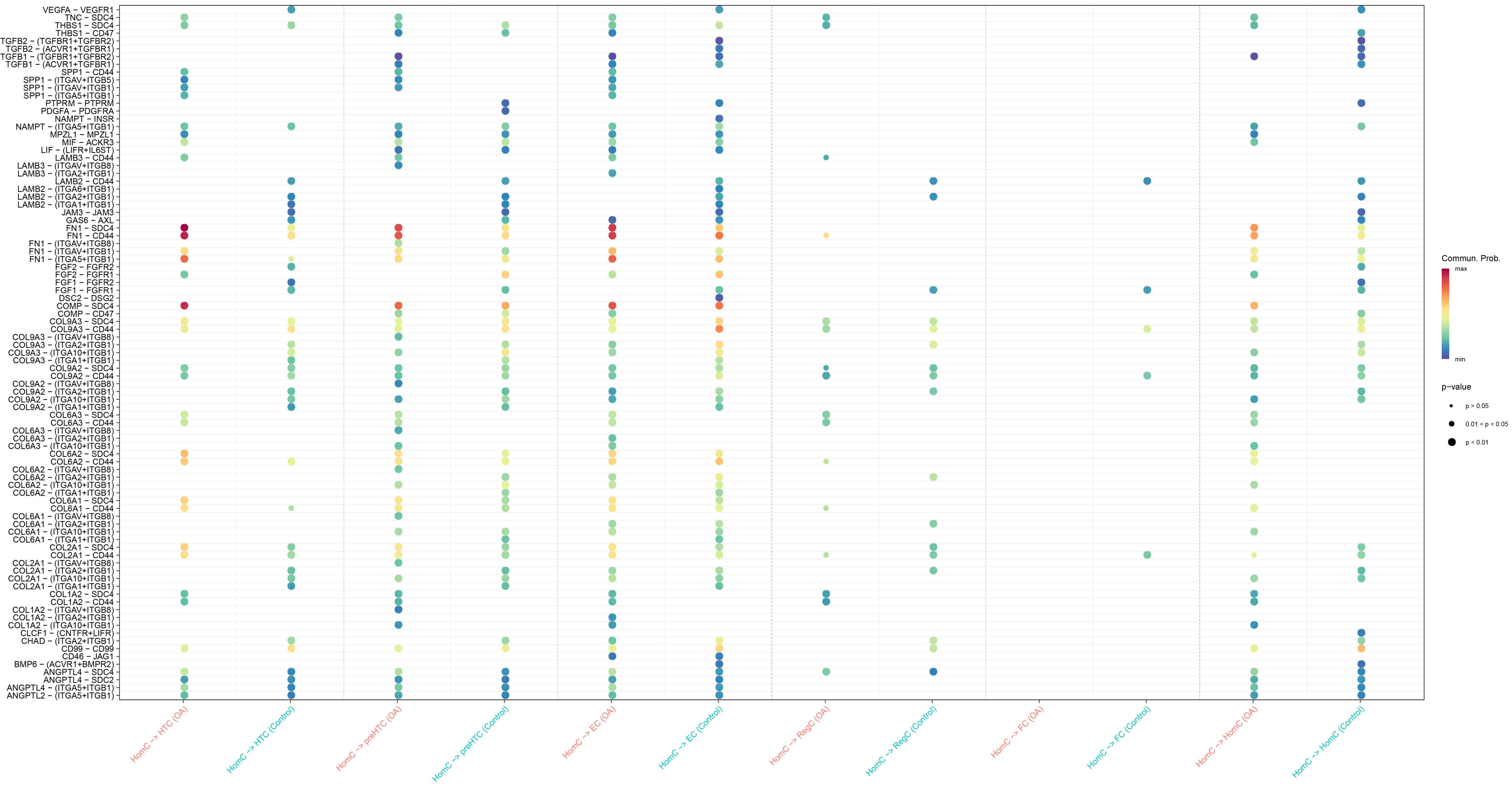

Supplement: Supporting Information 4 — Figure S3: Intercellular communication signals between HomC and other cell types in OA and normal tissues. The darker the color, the greater the probability of communication between cells. [file 1240226.f4.pdf]
